# Computational modelling for personalized transcatheter aortic valve replacement planning: a systematic review of complications and decision support

**DOI:** 10.3389/fdgth.2026.1832926

**Published:** 2026-06-04

**Authors:** Elisa Rauseo, Laura Bevis, Xu Chen, Steffen E. Petersen, Anthony Mathur, Gregory G. Slabaugh, Caroline H. Roney

**Affiliations:** 1 William Harvey Research Institute, NIHR Barts Biomedical Research Centre, Queen Mary University of London, London, United Kingdom; 2 Barts Heart Centre, Barts Health NHS Trust, London, United Kingdom; 3 Digital Environment Research Institute, Queen Mary University of London, London, United Kingdom; 4 School of Engineering and Materials Science, Queen Mary University of London, London, United Kingdom; 5 Department of Medicine, University of Cambridge, Cambridge, United Kingdom; 6 NIHR Barts Biomedical Research Centre, Queen Mary University of London, London, United Kingdom; 7 Centre for Cardiovascular Medicine and Devices, William Harvey Research Institute, Queen Mary University of London, London, United Kingdom; 8 The British Library, John Dodson House, Alan Turing Institute, London, United Kingdom

**Keywords:** computational simulation, decision support, digital twin, patient-specific modelling, personalized medicine, procedural planning, transcatheter aortic valve replacement

## Abstract

Patient-specific digital simulation is emerging as a tool to support personalized planning of transcatheter aortic valve replacement (TAVR), particularly as the procedure expands to younger, lower-risk patients, and more complex anatomies. Despite procedural advances, complications such as paravalvular leak, conduction disturbances, coronary obstruction, and aortic injury remain important determinants of outcome. Current pre-procedural planning relies heavily on computed tomography-based anatomical assessment, which is indispensable but largely static and cannot fully capture dynamic device-tissue interactions, and haemodynamic mechanisms underlying many procedural events. Computational modelling derived from patient-specific imaging can extend this assessment by simulating valve deployment, device-tissue contact, and flow, offering mechanistic insight and potential support for individualized procedural decision-making. This systematic review evaluates modelling approaches addressing TAVR complications and procedural planning, including high-risk scenarios such as bicuspid valves and valve-in-valve procedures. Across the literature, modelling enables patient-specific simulations and exploration of procedural strategies that may reduce complication risk. However, clinical translation remains limited by small study populations, heterogeneous methodologies, limited patient-specific validation, and lack of integration into routine workflows. Future progress will require validation against clinically meaningful endpoints, scalable digital infrastructure, and close collaboration between clinicians and engineers to incorporate simulation outputs into routine Heart Team decision-making.

## Introduction

Transcatheter aortic valve replacement (TAVR; also termed transcatheter aortic valve implantation, TAVI) has evolved from a therapy for patients with severe aortic stenosis (AS) at prohibitive surgical risk to a widely adopted intervention across a broad range of clinical profiles. Randomized trials and guideline updates have expanded its use to younger and lower-risk populations, increasing the importance of procedural safety and long-term outcomes (1–9). Real-world data mirror this evolution, with rapid growth observed among patients younger than 65 years in the United States, often driven by patient preference for transcatheter rather than surgical treatment despite limited long-term durability evidence (10). These trends heighten the need to ensure both procedural safety and long-term performance comparable to surgical aortic valve replacement (SAVR).

Despite technological advances, procedural complications remain key determinants of outcomes. Paravalvular leak (PVL), cardiac conduction abnormalities (CCA) requiring permanent pacemaker implantation (PPI), coronary obstruction, and aortic injury arise from complex interactions among device design, deployment strategy, and patient-specific anatomy (3, 11). These risks are further amplified in challenging scenarios such as bicuspid aortic valve morphology and valve-in-valve (ViV) procedures. Although pre-procedural decisions such as valve sizing, implantation depth, and whether coronary protection is needed are guided mostly by anatomical assessment and strongly influence complication risk, the mechanistic consequences of these decisions are not always fully predictable from imaging alone.

Multidetector computed tomography (MDCT) is the cornerstone of TAVR planning, providing detailed characterization of annular geometry, calcification, vascular access, and coronary anatomy. However, CT-based planning remains largely anatomical and does not capture dynamic processes such as device expansion, tissue deformation, and post-deployment haemodynamics, which are directly implicated in procedural complications (1, 2, 12).

Computational modelling has emerged as a complementary approach to extend CT-based planning by enabling patient-specific digital simulation of valve deployment, device-tissue interaction, and haemodynamic behaviour. By virtually reproducing implantation scenarios, these models can explore sealing behaviour, tissue contact patterns, and flow disturbances that are relevant to key procedural complications. For example, inadequate sealing may contribute to PVL, abnormal tissue contact may relate to conduction disturbances or tissue injury, and disturbed flow may be relevant to coronary obstruction and other haemodynamic consequences. In this way, they provide mechanistic insight into complication pathways and enable exploration of alternative procedural strategies, with the potential to enhance personalized decision-making in TAVR planning (10).

However, despite increasing methodological sophistication, translation into routine clinical practice remains limited. Across the literature, models differ in inputs and assumptions, target outcomes, validation methods, and reporting, limiting comparability and confidence for clinical use. Feasibility in real-world TAVR planning is also rarely addressed: many studies do not evaluate whether required data are available before the procedure, whether simulations can be performed within clinically relevant timeframes, how uncertainty should be communicated, or how outputs would influence Heart Team decision-making. These limitations highlight a gap between technical development and clinical applicability.

To date, modelling has been applied most extensively to procedural complications and pre-procedural planning, where translational potential is most immediate. Previous reviews have predominantly examined computational simulations in TAVR planning from methodological or outcome-prediction perspectives, emphasising model construction, numerical assumptions, and predictive performance (14–16). While these reviews are valuable for understanding technical development, they do not fully organise the literature around the complication-specific and planning questions that are most relevant to procedural decision-making. A complication-focused review is therefore needed to provide a clinically structured synthesis of the field, to show where modelling is most mature, and to identify the main gaps that still limit validation, comparability, and translation into practice.

Accordingly, this review adopts a multidisciplinary and clinically oriented perspective to evaluate computational modelling applications in TAVR. The aim is not to identify a single best modelling method for each complication, but to examine how different computational approaches have been applied across complication domains and planning scenarios, what insights they provide, and what currently limits their routine clinical use. We therefore focus on studies addressing procedural complications, high-risk anatomical scenarios, and pre-procedural planning, while also considering feasibility and broader barriers to clinical integration. In contrast, modelling addressing long-term TAVR degeneration and durability remains less mature and less directly applicable to peri-procedural decision-making, and is therefore beyond the scope of this review (13).

### Pre-procedural assessment and TAVR complications

Current guidelines recommend referring patients with severe AS to the Heart Team for evaluation (1, 2, 11). This multidisciplinary team provides treatment recommendations based on a comprehensive assessment. This includes the patient's clinical risk profile and frailty, technical considerations like the feasibility of TAVR and device choice, and the patient's preferences. While guideline indications inform when TAVR is appropriate, shared decision-making remains essential when selecting between TAVR and SAVR (1, 2, 11).

A comprehensive anatomical evaluation of the arterial vasculature and aortic valve complex, based on imaging, is required to determine suitability and the optimal vascular access route. Transthoracic echocardiography (TTE) is the first-line test for assessing AS severity, identifying coexisting valvular or ventricular disease, and monitoring prosthesis function and complications during follow-up (1, 17). MDCT is the gold standard for procedural planning due to its high spatial resolution. It is used to size the aortic annulus, assess vascular anatomy and access, and evaluate calcification distributions, sinus dimensions, and coronary ostia (12). This information is utilised to determine the best access method, device selection, and to estimate anatomy-related procedural risks. Advanced imaging modalities such as 3D echocardiography and 4D flow Magnetic Resonance Imaging (MRI) can provide additional structural and haemodynamic information when needed (17, 18).

Patient factors, including comorbidities, pre-existing vascular conditions, and procedure-related issues, influence post-TAVR outcomes (12, 19). Complications common to SAVR, such as infective endocarditis, valve thrombosis, and patient-prosthesis mismatch, can occur in TAVR, alongside TAVR-specific complications arising from the transcutaneous procedure or the device's interaction with anatomical structures. This includes: PVL, which may result from incomplete sealing and device malapposition; CCA, which may relate in part to implantation depth and contact with the membranous septum; coronary obstruction and risk of myocardial infarction, which depend on the relationship between the prosthesis and the coronary ostia; as well as aortic annulus rupture, stroke, vascular site complications, left ventricular perforation, cardiac tamponade, and the potential need for conversion to surgery (1, 2, 11, 20).

### Computational modelling techniques in TAVR

Several computational modelling approaches have been applied to TAVR to complement CT-based planning with a functional, mechanistic assessment of device-tissue interaction, and post-implant haemodynamics. In practice, modelling is most often used to explore procedural “what-if” scenarios (e.g., alternative device sizes or deployment strategies), interrogate mechanisms underlying complications, and generate quantitative outputs that may support planning in complex anatomies. At present, patient-specific modelling is used selectively, mainly in research settings and in a small number of clinically deployed platforms, because feasibility depends on the availability and quality of imaging inputs, the assumptions required to construct the model, turnaround time within the pre-procedural planning window, and the strength of validation evidence.

Modelling approaches vary in their degree of patient specificity. Idealised models use simplified geometries and model parameters based on physiological ranges or population averages, and can provide key insights into underlying physical mechanisms and help to identify important parameters. Patient-specific models, instead, incorporate individual patient data (most commonly CT-derived anatomy) and are intended to represent more realistic deployment and flow conditions. This is particularly relevant when anatomy falls outside typical population averages, for example in bicuspid aortic valve, heavy or asymmetric calcification, or after prior valve interventions such as ViV procedures. However, selecting appropriate patient-specific values requires careful interpretation of clinical measurements and modelling assumptions. In addition, segmentation, mesh generation, assignment of tissue and device properties, and specification of boundary conditions introduce practical constraints for clinical use, as they require substantial technical expertise and may involve operator-dependent choices that affect both turnaround time and the reproducibility of model generation and simulation outputs. For clinical credibility, models also require verification and validation against experimental benchmarks and, ideally, clinically meaningful endpoints.

Three main computational approaches are used in TAVR applications: finite element analysis (FEA), computational fluid dynamics (CFD), and fluid-structure interaction (FSI). FEA uses the finite element method to analyse the structural response of a structure to applied loads and boundary conditions by discretising complex geometries into finite elements and combining their behaviour to approximate the overall response, including the resulting deformation, stresses, and strains (21). FEA is most commonly used to simulate device crimping and deployment, frame expansion, and device–tissue contact. These simulations can estimate prosthesis frame deformation and apposition of the frame or sealing skirt to the native annulus and aortic root (relevant to sealing and PVL), stress distributions in the frame and surrounding tissue, and regions of high contact that may be relevant to conduction disturbance (22–26).

FEA requires information on device geometry and material properties and on the implantation site (patient-specific or idealised) including tissue properties. While FEA can provide useful insight into deployment mechanics and contact behaviour, it does not model flow and therefore cannot directly capture post-deployment haemodynamics. In addition, studies may simplify leaflet and skirt behaviour, and certain procedural steps (e.g., crimping) can be challenging to model robustly depending on numerical stability and assumptions (27, 28).

Computational Fluid Dynamics (CFD) is used to quantify post-implant haemodynamics by solving the governing equations of fluid motion (29). CFD can provide velocity, pressure, and shear-related metrics across the cardiac cycle and has been applied to study haemodynamic mechanisms relevant to PVL jets, flow disturbance around the frame, and markers associated with thrombosis risk, such as regions of flow stasis or recirculation (30, 31). CFD typically assumes fixed boundaries, meaning the patient-specific or idealised anatomy, the implanted valve frame, and the valve leaflets remain static during simulation. In addition to defining the geometry of the flow domain, CFD requires specification of fluid properties (e.g., density and viscosity) and prescription of boundary conditions that determine how blood enters and exits the model; these choices strongly influence predicted velocity fields, pressure gradients, regurgitant flow, and wall shear stress, and must therefore be selected carefully. Although CFD offers rich haemodynamic detail, it does not capture the coupled kinematic and dynamic interaction between blood flow and deforming valve leaflets; as a result, CFD studies often model the valve in a fixed open or closed state and may use idealised representations of the implanted device. In practice, boundary conditions are often approximated from limited clinical measurements, which can affect comparability across studies.

Fluid-structure interaction (FSI) couples FEA and CFD, enabling simulation of deformable, moving boundaries and their interactions with the flow (32). This is particularly useful for representing leaflet motion and the associated haemodynamic response more realistically than FEA or CFD alone. FSI can therefore provide additional insight into biomechanical factors affecting TAVR performance and longer-term device behaviour. Although geometric, material, and boundary-condition information is also required for FEA and CFD, FSI is more demanding because these inputs must be defined consistently across both the structural and fluid domains and coupled at the fluid-structure interface. This introduces additional coupling assumptions that do not arise when either domain is solved in isolation, and increases overall model complexity and computational cost. As a result, staged or simplified modelling approaches are commonly adopted (for example, sequential structural simulation of deployment followed by post-deployment flow or haemodynamic analysis, or FSI models in which the aortic wall is assumed rigid and only the leaflets are deformable) (22, 33–35) to balance physiological fidelity with computational feasibility.

**Figure 1:**
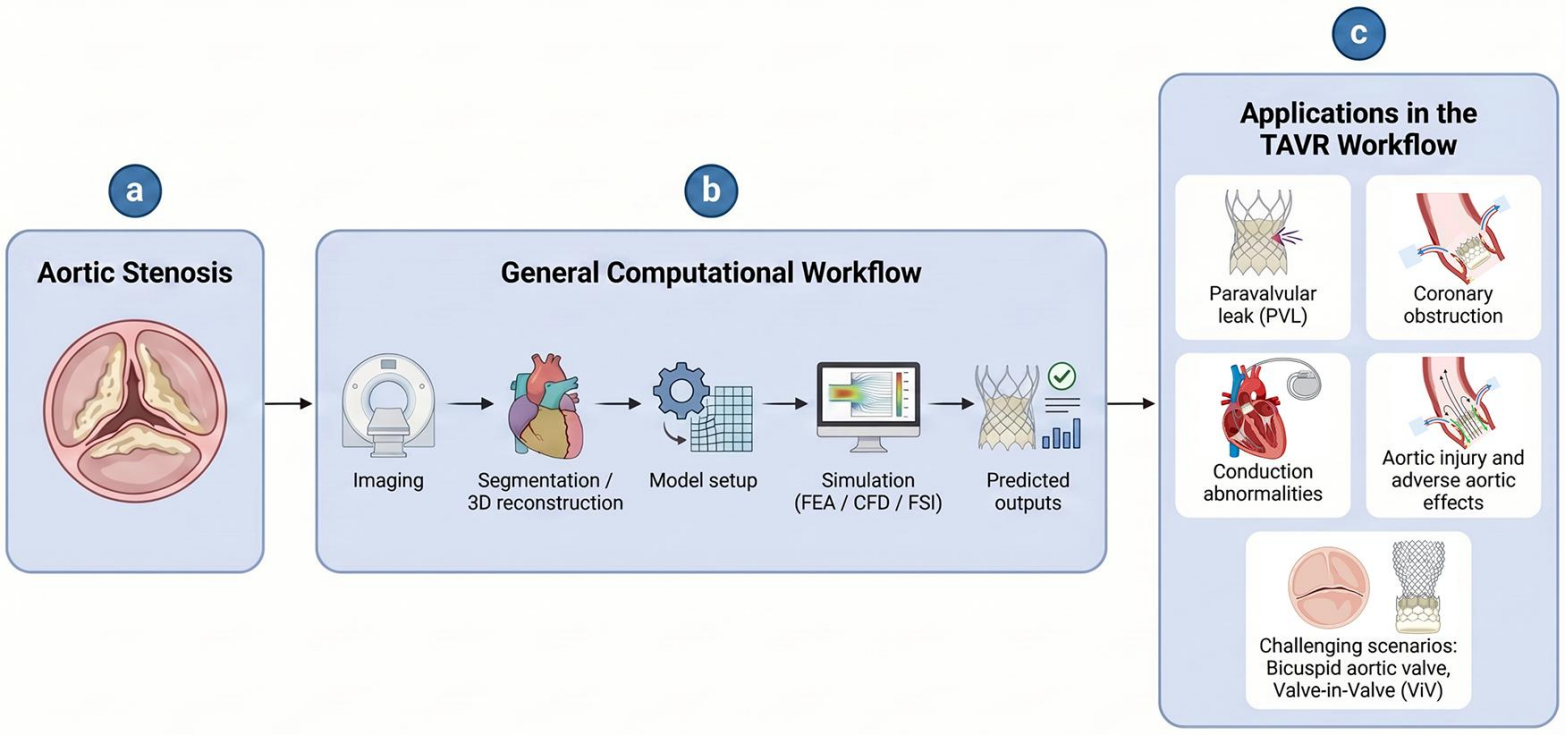
General computational workflow and main application areas of computational modelling in transcatheter aortic valve replacement (TAVR). Computational modelling can be used to support the TAVR workflow by linking pre-procedural anatomy to simulation-based evaluation of procedural risk and device performance. **(a)** Aortic stenosis anatomy represents the starting point for assessment. **(b)** Imaging data can then be used to generate a computational model through segmentation and three-dimensional reconstruction, followed by model setup and simulation using finite element analysis (FEA), computational fluid dynamics (CFD), or fluid-structure interaction (FSI), producing outputs relevant to procedural planning and risk assessment. **(c)** These outputs can be applied to major clinical questions in TAVR, including paravalvular leak (PVL), coronary obstruction, conduction abnormalities, aortic injury and adverse aortic effects, and challenging scenarios such as bicuspid aortic valve and valve-in-valve (ViV) procedures.

Together, these methods have been used in the literature to study mechanisms of TAVR complications, support pre-procedural planning, and inform device design. An overview of the applications of these methods in TAVR is provided in Table 1 and Figure 1. In this review, we focus on modelling studies related to procedural complications and planning, appraise their reported benefits and limitations, and highlight the barriers and future directions most relevant to clinical translation.

**Table 1:** Example applications of computational modelling in TAVR.

Method	Potential Applications for TAVR
Finite Element Analysis (FEA)	Complication mechanisms, planning, and procedural optimization: - Device anchoring, misalignment, and PVL - Structural correlates of aortic regurgitation risk (e.g., device-imposed tissue stress, stent deformation) - Aortic rupture - Stress-induced conduction abnormalities - Procedural steps e.g., crimping, balloon pre-dilation - Complex TAVR e.g., bicuspid aortic valve, ViV
Computational Fluid Dynamics (CFD)	Complication mechanisms and impacts, planning, and procedural optimization: - PVL jet flow - TAVR performance and associated flow-linked complications (e.g., thrombosis, aortic complications) - Impact of TAVR on coronary flow
Fluid-Structure Interaction (FSI)	More detailed insights into complex processes: - Dynamic behaviour of devices e.g., anchorage, PVL, and aortic regurgitation - Hydrodynamic performance - Aortic complications - TAV leaflet stresses and flow-leaflet interactions for TAVR performance

CFD, computational fluid dynamics; FEA, finite element analysis; FSI, fluid-structure interaction; PVL, paravalvular leak; TAV, transcatheter aortic valve; TAVR, transcatheter aortic valve implantation; ViV, valve-in-valve.

## Methods

### Search strategy

A systematic literature search was conducted from February 2024 to April 2025 across five databases (Web of Science, Scopus, PubMed, IEEE Xplore, and Engineering Village/Compendex), following Preferred Reporting Items for Systematic Reviews and Meta-Analyses (PRISMA) guidelines. The search was conducted independently by two researchers, one with a background in cardiology (E.R.) and the other in engineering and mathematics/fluid mechanics (L.B.). The most recent search was performed on April 4, 2025.

Search terms combined computational methods (“computational fluid dynamics”, “CFD”, “Fluid-structure interaction”, “FSI”, “computational simulation and analysis”, “computational modelling and analysis”) with TAVR -related terms (“bioprosthetic aortic valve”, “aortic valve prosthesis”, “aortic valve replacement”, “bioprosthesis”, “bioprosthetic”, “transcatheter aortic valve replacement”, “TAVR”, “transcatheter aortic valve implantation”, “TAVI”, “aortic valve prosthesis implantation”). Reviews, book chapters, conference abstracts, and preprints were excluded. Only articles published in English were included, with no restrictions on publication year.

The search strategy was intentionally broad, particularly for TAVR-related terms. This was necessary because many technically focused studies did not include the term “TAVR” or “TAVI” in their titles or abstracts; instead, they used more general terminology such as “bioprosthetic valve” or “aortic valve prosthesis,” but still referred to TAVR procedures. Likewise, we did not restrict the search by individual complication terms (e.g., “paravalvular leak”), as many relevant studies were broader in scope or did not explicitly name complications in their titles or abstracts. A narrower strategy would likely have excluded important papers. As a result, a large number of abstracts and full texts required manual screening for relevance.

### Eligibility criteria and study selection

We defined eligibility according to the clinical scope of this review, which focused on computational modelling applications relevant to TAVR procedural complications and pre-procedural planning. Eligible studies were original full-text articles published in English that used computational modelling approaches in the context of TAVR and addressed at least one of the following: (1) procedural complications affecting post-TAVR outcomes; (2) complex or high-risk anatomical scenarios associated with procedural complications, including bicuspid aortic valve and ViV procedures; or (3) pre-procedural planning questions directly relevant to minimising complications or improving procedural outcomes, such as device selection, sizing, implantation depth, release strategy, or deployment optimisation. This scope was chosen to focus on the area in which computational modelling currently has the most direct relevance to procedural decision-making and clinical translation.

We excluded studies not focused on TAVR, studies not using computational modelling, and studies describing modelling techniques without addressing a TAVR complication or clinically relevant planning question. We also excluded studies focused primarily on long-term post-implant valve performance (e.g., durability, thrombosis, structural valve degeneration), as these involve distinct methodological and validation considerations and fell outside the scope of this review. In addition, we excluded studies centred on device design or device comparison without analysis of specific procedural complications or planning applications.

Titles and abstracts were screened independently by both reviewers, with discrepancies resolved through discussion with a third reviewer (C.H.R.). The full texts of potentially eligible studies were then jointly assessed against the predefined eligibility criteria to determine final inclusion.

### Data extraction and synthesis

From each included study, we extracted the modelling approach (FEA, CFD, or FSI), the primary clinical application or complication addressed, the study scale, degree of patient-specificity, and validation approach. Degree of patient specificity was recorded descriptively, recognising that computational models ranged from idealised or synthetic anatomies to studies incorporating varying levels of patient-specific imaging or clinical data, rather than fitting a simple binary classification. Studies were then grouped according to their main focus, including PVL, coronary obstruction, conduction abnormalities, other post-TAVR complications, complex anatomical scenarios, and broader pre-procedural planning applications.

## Results

**Figure 2:**
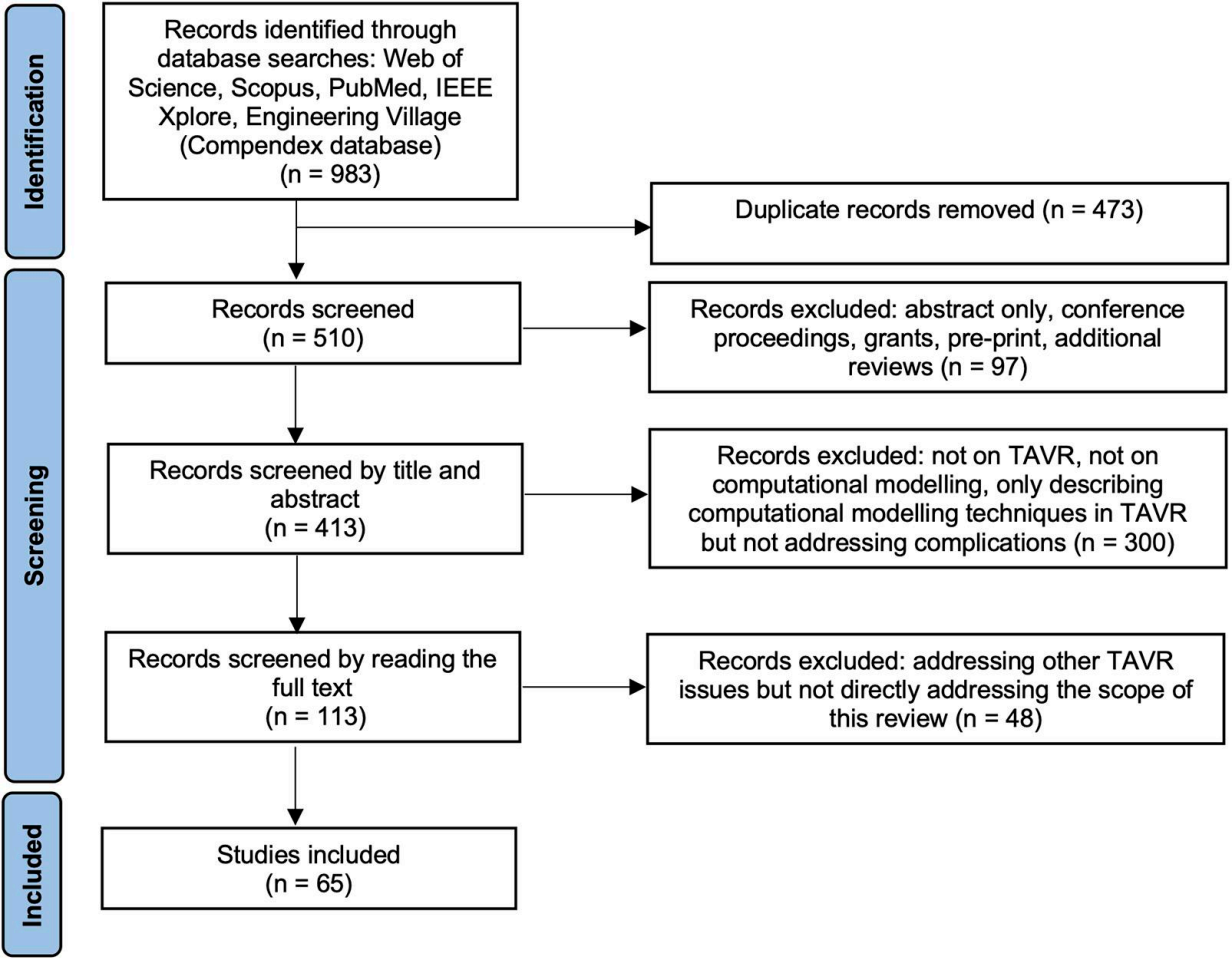
Study selection according to PRISMA guidelines.

The results of the study selection process are summarised in Figure 2. From 983 records identified across multiple database searches, 473 duplicates were removed, leaving 510 records for screening. An initial screening step excluded 97 records based on publication type (abstract-only publications, conference proceedings, grants, preprints, and reviews). The remaining 413 records were then screened by title and abstract, of which 300 were excluded because they were not focused on TAVR (e.g., addressing other valve prostheses or surgical procedures, the native aortic valve, other valves, or the aorta), did not use computational modelling, or described modelling techniques without addressing specific TAVR complications. A total of 113 full-text articles were assessed for eligibility. Of these, 48 were excluded because they focused on aspects outside the scope of this review, including studies on long-term post-implant valve performance (e.g., structural valve degeneration, thrombosis, durability), studies primarily supporting long-term device design, or those comparing devices (alone or versus SAVR) without analysing specific complications or planning questions.

The remaining 65 studies were included in this review. They were further classified according to their main focus: PVL (*n* = 21), coronary obstruction (*n* = 6), CCA (*n* = 3), other post-TAVR complications, including aortic issues (*n* = 8), and challenges in complex anatomical scenarios (*n* = 18). Finally, a group of studies (*n* = 9) investigated applications of modelling to support pre-procedural planning (e.g., selecting device type and size, or deployment strategies based on patient anatomy) or to optimise device performance with the aim of minimising complications.

A range of computational approaches was represented across the included studies (see Supplementary Tables S1–6). FEA was the most frequently used approach for structural analysis, and was primarily applied to simulate TAV deployment, frame deformation, device apposition, and tissue stress or strain, with relevance to complications such as PVL and conduction abnormalities. In several studies, FEA was used in a staged workflow in which the post-deployment geometry was subsequently used as input for CFD or FSI analyses to evaluate haemodynamics, including PVL-related jet flow and shear-related metrics. CFD analyses were used to characterise blood flow in the aortic root and across the valve, providing further insight into PVL mechanisms, wall shear stress (WSS), and disturbed or turbulent flow patterns in the aorta. One study additionally combined computational modelling with machine learning (ML) to improve outcome prediction (36).

Across the included studies, study scale was usually small, often limited to single-case or few-case simulations, although larger patient-specific cohorts were reported mainly in studies of PVL and challenging anatomical scenarios (Supplementary Tables S2 and S6). Patient-specific models predominated overall, but idealised, synthetic, and mixed modelling frameworks were also common, particularly in mechanistic or proof-of-concept studies. Validation approaches were heterogeneous and often limited or absent; when reported, they most commonly involved comparison with post-procedural imaging, echocardiography/Doppler or other haemodynamic measurements, or *in vitro* experiments, as summarised in Supplementary Tables S1–6.

## Discussion

Computational modelling in TAVR has developed rapidly over the past decade in response to persistent procedural complications and the expansion of the procedure to younger, lower-risk patients. Much of the literature is centred on pre-procedural planning and complication-focused applications, reflecting the need to move beyond purely anatomical assessment when procedural risk depends on device–anatomy interaction and post-implant haemodynamics. Although many studies used CT-based patient-specific anatomies, the field also includes idealised, synthetic, and mixed modelling frameworks, while the use of other imaging modalities such as MRI remains limited (31, 37).

The literature focuses mainly on complications and planning scenarios in which standard anatomical assessment may be insufficient to anticipate device–anatomy interaction. Paravalvular leak is the most frequently investigated complication, consistent with its prevalence and its close relationship with modifiable procedural factors such as device sizing, deployment strategy, and sealing behaviour. Computational modelling has also been widely applied in challenging settings, including bicuspid aortic valves and ViV procedures, where procedural risk is higher and standard planning is less established (38–40). In addition, some studies have explored broader applications such as device optimisation and simulation of alternative procedural strategies. While these applications are clinically relevant, they remain less directly linked to specific complication pathways, and are therefore summarised separately in Supplementary Table S1.

However, the maturity of the evidence varies considerably across complication domains. Heterogeneity across studies extends beyond modelling technique alone to include differences in study scale, degree of patient specificity, validation strategy, and reported endpoints. Together with small study populations, these differences limit direct comparison and help explain why translation into routine practice remains uneven. The following sections therefore examine the literature by specific complications and high-risk scenarios, where the current strengths, limitations, and translational potential of modelling can be assessed most clearly.

### Paravalvular leak

Paravalvular leak remains a major post-procedural complication, and is associated with increased morbidity and mortality (20, 41, 42). Modelling studies addressing PVL are summarised in Supplementary Table S2. Clinically, PVL is driven by incomplete sealing and non-uniform device expansion, often related to annular/root geometry, calcification distribution, device design, and deployment strategy (41, 42). Computational modelling is well-suited to interrogate these mechanisms because it can link procedural choices (e.g., implant depth, balloon volume/pressure, post-dilatation) and anatomical features (including calcification patterns) to frame deformation/apposition and to PVL jet haemodynamics. In general, FEA-based approaches infer PVL risk from gaps between the frame/skirt and native tissue, whereas CFD/FSI approaches estimate PVL jet flow patterns and their haemodynamic consequences (43–45). Incorporating patient-specific anatomies, calcification patterns, and device characteristics (e.g., size, material properties, stent design, orientations) may improve realism of PVL estimate and, where evaluated, agreement with post-procedural imaging, potentially informing device selection and positioning strategies to mitigate PVL risk (22, 43, 46).

Across studies, one consistent message is that PVL is highly sensitive to modifiable deployment variables, particularly implantation depth, ballooning strategy, and valve position (22, 43, 47). For example, Bianchi et al. (22) combined FEA and CFD in three patient cases to examine implantation depth and balloon inflation volume, and reported good agreement between predicted PVL location/volume and post-procedural echocardiography, while also evaluating haemodynamic and thrombogenic metrics. They found that a higher, more aortic implantation position (i.e., a shallower ventricular implantation depth) reduced PVL volume by up to 47%. Ghosh et al. (43) used FSI to assess hydrodynamic performance and leaflet stress in simulated anatomy, reporting that lower ventricular deployment minimised PVL, device migration and thrombogenicity. Similarly, Meng et al. (47) proposed a rapid numerical framework to simulate self-expanding valve deployment at different positions in patient-specific anatomies, including bicuspid and tricuspid valves. The model, validated against clinical outcomes, linked implantation depth to both PVL and atrioventricular conduction block, highlighting how simulation can guide valve choice and release strategy to reduce procedural risks. These studies illustrate how modelling can support scenario testing around positioning and deployment, and how a single procedural parameter may affect multiple complication pathways.

Multiple studies emphasised that realistic patient-specific aortic root geometry, including native leaflets and calcification distributions, alongside precise stent geometry reconstruction, are key determinants of simulated sealing and PVL estimates. Morganti et al. (23) demonstrated through FEA that stent apposition alters anatomical conﬁgurations and stress distributions on the aortic wall, and that frame-to-wall distance metrics aligned with clinical data for PVL prediction. Building on this, a recent study (27) presented a numerical framework simulating the full crimping and deployment of TAVR devices in patient-specific aortic roots, including native leaflets and calcifications, validated against experimental results. The authors showed that appropriate balloon inflation pressure is critical to achieve sufficient flow area and effective stent-root contact, while minimising deformation and reducing PVL risk. Together, these studies support a clinically intuitive concept: PVL is not only a sizing issue, but also a deployment and anatomy-device interaction issue. Modelling can integrate procedural parameters with patient-specific anatomy to explore strategies that may reduce PVL risk.

Calcification pattern has been explored as a mechanistic driver of PVL variability. Luraghi et al. (44) used an FSI model to compare calcification distributions and reported different PVL severities depending on calcification location, with cusp coaptation calcifications causing mild PVL, and attachment line calcifications leading to moderate PVL. Spadaccio et al. (48) combined biomechanical modelling with CT imaging to illustrate how calcification-induced device misalignment and stent deformation result in adverse outcomes like PVL. Prisco et al. (49) highlighted that increasing the area occupied by the native valve significantly reduces post-operative regurgitation, emphasising the native valve's role in mitigating PVL.

Computational modelling has also been used to study PVL's impact on cardiovascular structures and its association with other complications. Long-term PVL, even if mild, can lead to vascular issues, increasing risks of stroke, arrhythmias, and coronary ischaemia due to haemodynamic changes (45, 50, 51). FSI analyses assessing the haemodynamic effects of PVL on aortic flow have demonstrated that PVL in TAVR increases blood velocity, pressure drop and WSS, potentially leading to flow recirculation, thrombus formation and aortic wall damage (52). Smaller or undersized valves exacerbated these effects (45, 51).

Patient-specific simulations have been also explored for risk stratification. For instance, Dowling et al. (30) conducted a multi-centre retrospective study demonstrating that patients predicted to have significant PVL (>16.0 mL/s) via CFD simulations had a higher risk of death within two years compared to those with no significant predicted PVL, supporting the potential clinical value of simulation-derived PVL metrics as an adjunct to standard planning while also highlighting the need for broader, standardised validation across centres and devices.

Overall, PVL is the complication in which modelling currently appears closest to clinical utility, because it integrates anatomy, deployment mechanics, and haemodynamic consequences in a way that directly maps onto procedural planning, although broader standardised validation is still required.

### Coronary obstruction

Coronary obstruction is an uncommon but potentially devastating complication of TAVR, typically occurring during or immediately after valve deployment, and requiring urgent intervention; delayed obstruction has also been reported and is associated with adverse outcomes (53–55). The main mechanism is the displacement of the calcified native leaflets towards the coronary ostia, with consequent obstruction of the coronary flow. Alternatively, displaced leaflets can obstruct the coronary sinuses by apposition against the sinotubular junction, functionally “sealing” the sinus and limiting coronary filling (53–56).

The risk of coronary obstruction depends on a complex interplay of patient characteristics, device, and procedural factors. Low coronary heights (defined as the distance from the coronary ostia to the aortic annulus), small sinuses of Valsalva, female sex, balloon-expandable (BE) devices, and ViV procedures are recognised risk factors (53, 54). Pre-procedural identification of high-risk patients is therefore central to planning. Cardiac CT is generally used for identifying high-risk patients, especially when left coronary artery height is < 12 mm and/or a sinus of Valsalva diameter is < 30 mm (54). However, CT-based measures may not fully capture the dynamic leaflet–frame interaction and post-deployment geometry that determine coronary compromise, which can limit predictive accuracy in borderline cases.

Modelling studies investigating the risk of coronary obstruction are summarised in Supplementary Table S3. Patient-specific computational modelling can extend CT-based assessment by simulating valve deployment and leaflet displacement within image-derived anatomy. For instance, Fan et al. (57) used FE simulations based on pre-procedural CT to model valve deployment in fourteen patients undergoing TAVR, including those with coronary obstruction, at high risk, and at low risk, incorporating native calcified leaflets and their interaction with the frame. Predicted post-deployment frame deformation closely matched post-procedural imaging, validating the approach. They also reported that shorter simulated coronary-structure distances (leaflet, calcium, or frame) correlated with obstruction risk, highlighting leaflet displacement as a frequent cause. Importantly, the simulated coronary-stent distance was proposed not only as a predictor of intra-procedural obstruction but also as a potential surrogate marker for future coronary access, a key consideration as TAVR expands to younger patients. Despite the small cohort and omission of other anatomical or flow parameters, this study illustrates the potential of simulations to refine risk stratification and procedural planning beyond CT alone. Similarly, Heitkemper et al. (58) suggested that the native cusp-to-coronary ostium distance more accurately estimates obstruction risk than traditional CT measures.

Beyond risk prediction, computational studies have explored how deployment strategy and anatomy influence coronary flow and access (59–61). For instance, Kandail et al. (61) reported that supra-annular deployment may improve coronary flow in their simulations, while also altering WSS patterns, influencing aortic dilation and pro-atherogenic alterations in coronary arteries. Oks et al. (59) showed that commissural misalignment can impair coronary perfusion and valve functionality, underscoring the procedural importance of axial/rotational control during deployment. Scuoppo et al. (60) used FEA to show that higher implantation depth and undersized second TAV devices in ViV procedures reduce coronary flow, guiding optimal device placement and sizing to prevent compromised coronary access, especially in complex anatomies.

FSI-based models have also been used to characterise coronary flow dynamics after TAVR, providing insights into long-term mechanisms of coronary obstruction. Wald et al. (62) used a simplified 2D FSI model to propose a haemodynamic mechanism by which valve leaflet dynamics influence coronary blood flow, linking orifice area, jet velocity, vortex location near the coronary ostium, and diastolic pressure gradients. Their model offered a potential mechanistic explanation for the clinical observation that resting coronary blood flow is elevated in AS and declines toward normal after TAVR, and highlighted how valve design and size may affect long-term coronary perfusion.

In practical terms, the main added value of modelling in this domain is to refine risk assessment when static CT measurements leave residual uncertainty about post-deployment leaflet–frame relationships or future coronary access. However, the evidence base remains heterogeneous, and stronger validation across device types and clinically meaningful endpoints is required.

### Cardiac conduction abnormalities

The interaction between the TAV device and native left ventricular outflow tract can affect the conduction system located below the membranous septum (MS), leading to CCA, and, in some patients, the need for PPI. This risk depends on device design, implantation depth, and patient-specific anatomy, and may be accentuated in bicuspid anatomies, where structural differences may further influence device–septal interaction (63).

Anatomically, measuring the MS length, the distance between the aortic annulus plane and the expected course of the His bundle, has been associated with PPI risk, with shorter membranous septum length linked to higher risk (64, 65). However, translating these anatomical markers into actionable procedural strategy remains challenging, in part because precise localisation of the His bundle region is not directly available during the procedure.

Modelling studies focused on CCA are summarised in Supplementary Table S4. Patient-specific simulations of valve deployment can, in principle, support risk assessment by estimating device–tissue contact, stress/strain distribution in regions relevant to the conduction system, and the impact of modifiable procedural factors such as implantation depth and sizing. For instance, Reza et al. and Bosi et al. (24, 25) showed that higher strain in the MS region (used as a surrogate for conduction tissue vulnerability) was associated with conduction disturbance and potential need for PPI. McGee et al. (26) highlighted the influence of implantation depth on stress distribution, emphasising the importance of valve positioning in reducing conductance interference risk.

By providing insights into biomechanical factors such as stent deformation, implantation depth, and strain distribution, computational models offer clinicians a valuable tool to optimise personalised procedural planning, mitigate the risk of CCA, and improve patient outcomes in TAVR procedures. However, the variability across patients and the limited number of studies on this topic suggest that further computational analysis on larger patient cohorts is needed before modelling outputs can be used reliably to guide routine decision-making.

### Other TAVR procedural complications

Compared with PVL, coronary obstruction, and conduction disturbances, modelling studies on other procedural complications remain fewer and more exploratory, but they illustrate the broader capacity of simulation to interrogate adverse device–anatomy and haemodynamic interactions (Supplementary Table S5).

Although infrequent, aortic injuries post-TAVR, including aortic annulus rupture, dissection, and fistulas, are clinically serious, and are associated with anatomical risk factors such as severe calcification and eccentric or asymmetric aortic root geometry (66). In this context, computational modelling has been used to explore how post-implant geometry and flow may contribute to adverse aortic loading. For example, Wen et al. (67) used CFD to show that elliptical annulus shapes after TAVR can disturb ascending aortic haemodynamics, with regions of low time-averaged wall shear stress (TAWSS) alongside elevated oscillatory shear index (OSI) and cross-flow index (CFI) observed in the ascending aorta, suggesting a potential mechanism by which altered post-implant geometry could contribute to adverse aortic conditions. More broadly, CFD-based analyses have been used to characterise how TAVR alters aortic flow patterns and haemodynamic stresses that may be relevant to aortic complications (31).

Anchoring and device stability represent another procedural concern. Wu et al. (68) used FSI simulations to examine anchoring behaviour, highlighting the role of radial force and frictional interaction in resisting migration. Beyond migration, malposition or device-anatomy interaction can also contribute to adjacent-structure complications, including mitral valve interaction or worsening mitral regurgitation, underscoring the importance of deployment strategy and positioning.

Finally, computational studies, especially based on CFD analyses, have explored embolization-related mechanisms and potential approaches to cerebral protection, primarily by modelling flow and particle transport during or after valve deployment (69). Another study employed CFD to analyse circulatory changes after the procedure to assess bleeding risk. However, clinical validation and endpoint correlation are necessary before this tool can be used to guide personalised post-operative care to minimise complications and enhance outcomes (70).

Although these applications are less mature, they show that modelling can generate hypotheses and mechanistic insight in areas where conventional planning provides limited functional information.

### Planning TAVR in challenging scenarios

Complex anatomies are where simulation-based planning may have the highest practical value, because standard CT-based rules are often less established and procedural uncertainty is greater (Supplementary Table S6).

The high prevalence of bicuspid aortic valve in younger patients, coupled with the expanding use of TAVR in this group, highlights the need for strategies to prevent complications in this subgroup (40). This condition presents unique challenges, such as eccentric annulus, calcification patterns, aortic dilatation, and asymmetric jet flow, contributing to a higher risk of complications, like PVL (71–76), thrombosis (77, 78), and conduction disturbances (79). Additionally, asymmetric flow and altered leaflet/frame mechanics may increase local stresses and potentially influence longer-term device behaviour, although outcome-level durability data remain limited. Computational modelling can help interrogate these mechanisms and support planning decisions by linking anatomy and calcification patterns to device deformation, sealing, and deployment behaviour (71, 72). For instance, Zhang et al. (28) reported that anatomical morphology and calcification volume influenced balloon behaviour during deployment, suggesting that simulation may help explore balloon selection and sizing strategies in this subgroup.

Another important challenge in bicuspid anatomy is the frequent coexistence of ascending aortic dilation, which often limits the indication for TAVR because of concerns about accelerated aortic growth. CFD has been used to explore this scenario, providing insight into the haemodynamic environment in a bicuspid aortic valve with dilation. In a small study, An et al. (80) reported abnormal vortical and helical flow with elevated WSS in the ascending aorta before TAVR, with the altered WSS pattern linked in the broader literature to progressive dilation and dissection risk. Following TAVR, these disturbances and WSS magnitude were reduced, suggesting the procedure may not worsen, and could potentially improve, the haemodynamic drivers of aortopathy, and supporting its potential feasibility in these complex cases, although the impact on long-term aortic growth and dissection requires further study.

Beyond bicuspid anatomy, computational modelling has also been applied to other complex settings where anatomy or comorbidity may complicate standard CT-based planning. Coexisting structural or ventricular disease can alter haemodynamics and mechanical interaction in ways that may influence procedural performance (81). CFD modelling can provide patient-specific insights, helping to assess feasibility and predict procedural performance. For instance, Khodaei et al. (82, 83) developed a framework using non-invasive patient data to quantify flow-derived metrics, potentially supporting risk assessment in selected complex cardiovascular conditions. Modelling has also been used to simulate procedures in challenging morphologies, such as pure non-calcified aortic regurgitation, ViV implantation in failed bioprostheses, or TAVR after prior aortic root replacement, where anticipating device–anatomy interaction may help inform device selection, sizing, and positioning to minimise complications (84–86).

Overall, these studies suggest that the primary value of computational modelling in this setting lies in anatomies where standard CT-based planning is insufficient to predict device-anatomy interaction. In such cases, patient-specific simulation can provide additional insight into deployment behaviour, sealing, and flow patterns, supporting more informed decisions on device selection, sizing, and implantation strategy. However, current evidence remains limited to small cohorts and heterogeneous methodologies, and further validation against clinically meaningful outcomes is required before routine integration into practice.

### Clinical implications, limitations, and future directions

Computational modelling has the potential to complement CT-based planning by simulating patient-specific “what-if” scenarios to inform device choice, implantation strategy, and procedural decision-making. These capabilities may be particularly valuable in younger patients, in whom even minor complications, such as mild PVL, conduction disturbances requiring PPI, or difficulties with coronary access, need to be minimised as they can accumulate into substantial long-term consequences (39). By linking anatomy and procedural choices to deployment mechanics and post-implant haemodynamics, modelling may help clarify complication mechanisms and support more tailored planning in selected cases, with the broader aim of improving procedural safety as TAVR is considered alongside SAVR in younger individuals.

As TAVR expands to more complex scenarios, including bicuspid aortic valves and ViV procedures, the potential value of simulation-based planning becomes more apparent. For instance, ViV interventions are expected to increase as younger patients outlive their initial bioprosthesis. Although generally feasible, these procedures can be challenging due to several anatomical or device-related factors, leading to increased risk of complications such as coronary obstruction, and sinutubular sequestration (38, 39). Computational modelling can be particularly valuable in these settings, by simulating patient-device interaction and exploring alternative device and deployment strategies to minimise complication risk (60, 84–86). Early clinical evaluation suggests potential utility in selected cases. In the prospective multicentre observational PRECISE-TAVI study, CT-derived simulation used as an adjunct to standard CT planning in challenging anatomies was reported to influence Heart Team planning decisions, including valve size and target implantation depth. The simulation also provided additional decision support beyond CT alone, including markers related to PVL risk, and regions of higher contact relevant to conduction disturbance (87). However, the available evidence remains observational, and the outcomes reported to date have focused on the influence of modelling on procedural planning decisions rather than on downstream clinical endpoints. Randomised trials demonstrating improved patient outcomes with simulation-guided planning are therefore still needed before routine implementation can be recommended.

Valve durability represents a key consideration in TAVR planning, particularly when evaluating treatment strategies in younger patients and in the context of TAVR versus SAVR. However, modelling in this domain differs from peri-procedural modelling in its aims and timescales. Rather than simulating acute deployment outcomes, durability modelling aims to characterise long-term processes such as cyclic leaflet stress, fatigue, and progressive tissue remodelling, whose clinical consequences manifest over years and cannot yet be validated within timescales relevant to procedural planning. The underlying computational methods (FEA, CFD, FSI) are the same, but their application is currently less directly transferable to peri-procedural clinical decision-making. Durability models typically rely on long-term assumptions and surrogate biomechanical or haemodynamic markers to infer structural valve degeneration, with limited validation against long-term clinical outcomes. A recent systematic review (13) suggests that these approaches face substantial challenges in linking simulation-derived metrics to clinically meaningful endpoints. As a result, although durability is central to treatment selection, current modelling frameworks are not yet able to provide robust, patient-specific decision support in this context. With further methodological development and the availability of long-term clinical data to validate surrogate markers, modelling may, in the future, support more informed durability assessment and contribute to treatment selection.

Despite the potential of computational modelling to support TAVR complication assessment and planning, several limitations still restrict immediate clinical adoption. Many studies rely on idealised or synthetic models, whereas patient-specific studies are often limited to small cohorts, and therefore represent only a restricted range of anatomies and devices, limiting generalisability. For model credibility, models need careful technical verification and validation against experimental benchmarks and clinical data. However, validation is inconsistently performed across studies and is often limited to post-procedural imaging-based endpoints rather than long-term clinical outcomes. Additionally, heterogeneity in modelling assumptions, reported outputs, validation strategies, and performance metrics further limits cross-study comparison and makes it difficult to accumulate consistent evidence for practice. Finally, the lack of standardised verification procedures and uncertainty reporting remains a barrier to model credibility and reproducibility across centres (88). Addressing these gaps, through larger multi-centre, multi-device datasets, prospective validation against clinically meaningful endpoints, and systematic treatment of uncertainty, will be necessary before modelling can be relied upon for routine clinical decision-making.

Even when models are technically feasible, implementation constraints can limit clinical use. Patient-specific pipelines can be time- and resource-intensive, and turnaround time may not reliably fit within the pre-procedural planning window without automation and adequate computational resources. While incorporating GPU or High-Performance Computing can reduce runtime, the overall workflow still depends on high-quality imaging inputs, segmentation and model preparation, parameter selection, and robust quality control. In addition, limited access to large, well-curated imaging and clinical datasets restricts model development and external validation across diverse populations and devices. Integration with hospital IT systems also poses challenges, including interoperability, governance, and data security considerations.

Several methodological advances may reduce barriers. For example, the use of reduced-order models (ROMs) and machine and/or deep learning approaches can accelerate specific tests, or emulate components of high-fidelity simulation, helping to bring runtimes closer to clinically acceptable timeframes (89–91). However, as of yet, these methods are still in development and cannot capture the complex small-scale dynamics of TAVR without some level of advanced biophysical simulation. Methods such as statistical shape modelling (SSM) can be used to generate synthetic datasets to overcome limited data availability (89). While the use of these methods in TAVR remains limited, these approaches hold promise for addressing key barriers to clinical translation by making patient-specific modelling faster, more scalable, and better integrated into routine planning. Integrating artificial intelligence (AI) into the computational modelling pipeline can help make complex simulations more clinically practical. For instance, AI can automate tasks like image segmentation and anatomical measurement, speed up the preparation of geometries used for simulation, predict outcomes such as pressure gradients or valve areas, and simplify workflows involving large datasets. These advances can reduce time, manual effort, and variability, which are crucial for clinical adoption of modelling tools. However, many of these AI-assisted tools are still at the proof-of-concept stage and require validation. Further work is therefore needed to ensure reproducibility, interpretability, and integration into clinical care.

Looking ahead, the ultimate promise of computational modelling in TAVR is the creation of patient-specific TAVR digital twins, which are virtual replicas of a patient's cardiac anatomy and physiology that incorporate multimodal imaging and clinical data, continuously updated as patient conditions change. These twins could simulate TAVR procedures in real time, enabling personalised and more precise treatment strategies, while addressing variability in patient responses (92). However, realising this promise will require stronger validation against clinically meaningful endpoints, standardised reporting of assumptions and uncertainty, and workflow designs that fit routine Heart Team decision-making. Progress will depend on sustained clinician-engineer collaboration so that modelling targets clinically relevant questions, uses appropriate validation standards, and produces outputs that can be interpreted and acted upon in practice.

### Strengths and limitations of this review

The main strength of this review is its multidisciplinary perspective, combining expertise in both modelling and cardiology to evaluate the literature across technical and clinical domains. By bridging engineering and clinical viewpoints, it provides a balanced appraisal of how computational modelling may inform procedural planning and support performance prediction in TAVR, recognising that most reviewed studies remain at the stage of feasibility or proof-of-concept rather than demonstrating a direct impact on clinical outcomes. This perspective may also help improve communication between engineering and clinical communities and support more clinically interpretable future research.

Despite a broad search across multiple databases using extensive keywords, some relevant studies might have been missed, especially some engineering-focused papers that did not explicitly reference “TAVR” or proxy terms in their titles or abstracts. Nevertheless, the studies included adequately addressed our research questions and are consistent with those recognised in prior reviews (14–16).

Finally, we did not perform a meta-analysis because the included studies were highly heterogeneous at several levels. Heterogeneity was present not only in computational framework (FEA, CFD, FSI), but also in the degree of patient specificity, study scale, procedural scenarios modelled, and validation strategy. Reported endpoints also differed substantially, ranging from mechanistic outputs such as stress, deformation, or regurgitant flow to surrogate imaging-based measures and clinical outcomes. In addition, studies used different boundary conditions, material properties, and assumptions regarding device deployment and tissue behaviour. Many were based on only a few patient-specific cases or on idealised anatomies. Together, these differences limited direct comparability and made it impossible to derive a common effect size or perform a meaningful pooled analysis. We therefore adopted a narrative synthesis, which was better suited to describing the capabilities, limitations, and translational potential of the literature. Future work would benefit from more standardised reporting of modelling assumptions, validation approaches, and outcome definitions, which may enable more robust comparative analyses.

## Conclusions

This complication-focused systematic review shows that computational modelling in TAVR has reached different stages of maturity across application domains. The evidence is currently most developed for PVL, while applications in coronary obstruction, conduction abnormalities, and complex anatomies remain promising but less mature. Overall, modelling appears most useful in complex or borderline cases where standard CT-based assessment alone may be insufficient to anticipate device–anatomy interaction, complication risk, or post-implant haemodynamic behaviour. However, broader clinical translation remains limited by small study populations, methodological heterogeneity, and inconsistent validation. Future progress will require prospective studies linked to clinically meaningful endpoints, scalable workflows that can be integrated into real-world Heart Team decision-making, and close collaboration between clinicians and engineers to ensure that modelling approaches address clinically relevant questions and generate actionable, interpretable outputs.
