# Supplementary material for: Computational modelling for personalized transcatheter aortic valve replacement planning: a systematic review of complications and decision support
Source: Front Digit Health. 2026 Jun 4;8:1832926. doi: 10.3389/fdgth.2026.1832926 (PMC13275269; doi:10.3389/fdgth.2026.1832926)
Supplement: Supplementary file 1 [file Datasheet1.pdf]

Supplementary Materials

1. **Supplementary Table 1: Computational modelling studies to optimise TAVR performance and minimise overall complications.**

2. **Supplementary Table 2: Mechanisms linked with paravalvular leak in TAVR.**

3. **Supplementary Table 3: Impact on coronary flow and risk of coronary obstruction with TAVR.**

4. **Supplementary Table 4: Factors involved in cardiac conduction abnormalities post-TAVR.**

5. **Supplementary Table 5: Other procedural TAVR complications.**

6. **Supplementary Table 6: Planning TAVR in challenging scenarios.**

7. **Supplementary References.**

Supplementary Table 1: Computational modelling studies to optimise TAVR performance and minimise overall complications.

Authors	Year	Degree of patient specificity	Computational Approach	Main findings	Study scale/Sample	Validation approach
Optimising device design						
Carbonaro D. et al [1]	2023	Idealised	FEA	Material model parameters of simulated TAV frames significantly influence TAV mechanical performance in terms of device anchorage and peak stress. TAV frame geometry and aortic root anatomy have minimal impact on these parameters.	3 idealised aortic root anatomies, with 2 different TAV frames	Comparison of predicted metrics from surrogate models, describing approximate relationship between TAV material parameters and FE outputs, against simulated biomechanical quantities. No comparison with clinical/real-world data.
Carbonaro D. *et al* [2]	2021	Idealised	FEA	Present an efficient optimisation framework for TAV frame geometries, enhancing mechanical performance by reducing pullout force, peak aortic root stress, and contact pressure, tailored to various aortic root anatomies.	1 idealised aortic root model, with and without 2 patterns of idealised calcification deposit distributions based on previous studies in aortic stenosis patients	Comparison of predicted metrics from optimisation with values simulated from optimal case. No comparison with clinical/real-world data.
Gessat M. *et al* [3]	2014	Patient-specific	FEA	FEA analysis of TAV stents segmented from post-TAVR CT images to estimate the radial force	21 patient-specific cases, each with their clinically	Accuracy of automated stent detection and reconstruction from CT images compared with manually identified stent

				imposed on the aortic tissue by the device, potentially aiding in understanding complications and improving stent design.	implanted TAVR device	landmarks in 5 cases. Simulated vs actual deformation compared in experimental loading test in vitro. Comparison of hoop forces with range obtained in previous study.
Optimising procedural planning						
Mutlu O. *et al* [4]	2025	Patient-specific	FEA	Patient-specific FEA of TAVR with different valve sizes showed that aortic geometrical variations and TAV size strongly influence wall stresses and deformations, highlighting the importance of virtual size testing in borderline anatomies.	14 patient-specific anatomies with parameterised model of native aortic leaflets tuned to patient dimensions, each with 3 sizes of 1 TAVR device	None
Morany A. *et al* [5]	2024	Idealised / parametric	FSI + FEA (multiscale)	Multi-scale FSI–FE models compared fibrocalcific (FAS) versus calcific (CAS) aortic stenosis and post-TAVR outcomes, showing CAS led to higher contact pressure on the aortic wall, lower anchoring area, and higher paravalvular leakage than FAS, despite a larger opening area, highlighting the role of underlying tissue	Parametric multi-scale aortic valve models representing pathology-free, fibrocalcific (FAS), and calcific (CAS) tissue archetypes, plus a fourth TAVR post-procedure model.	None

				pathology in TAVR outcomes.		
Li J. *et al* [6]	2022	Patient-specific	FSI	Compare outcomes of TAVR using different prosthetic valves, with implications for prosthesis selection and postoperative prediction: balloon-expandable valves showed better circular cross sections, smaller paravalvular gaps, and superior hemodynamic parameters compared to self-expanding valves, which had lower aortic stress and stroke risk.	1 patient-specific anatomy including patient's native leaflets and calcifications, with 2 different TAVR devices	None
Govindarajan V. *et al* [7]	2022	Patient-specific	FSI	Accurately predict the haemodynamic performance of TAVR, significantly reducing turbulent flow and energy dissipation compared to stenosed valves and aiding in optimal valve size and design selection.	1 patient-specific anatomy of left ventricle and ascending aorta, with implanted TAVR device	Comparison with pre- and post-TAVR clinical data (echocardiography/CT).
Bongert M. *et al* [8]	2018	Patient-specific	FSI	FSI simulations using MRI data reveal different stress patterns and movement behaviours in valve leaflets, aiding in the selection of appropriate	1 patient-specific anatomy with 2 undeformed, idealised TAVR devices merged to	None

				aortic valve therapy by incorporating individual anatomy into pre-operative planning.	patient-specific aorta	
Russ C. *et al* [9]	2013	Patient-specific	FEA	Investigate the impact of calcification location and size on TAVR outcomes to guide accurate, patient-specific device positioning and inform stent design adaptations.	1 patient-specific anatomy including patient's native leaflets and calcifications, with and without calcifications, with 1 TAVR device.	Simulated TAVR stent deformation compared with real deformation observed in post-TAVR CT scans.

Modelling studies addressing more generally TAVR performance summarised in Supplementary Table 1: [1], [2], [3], [4], [5], [6], [7], [8], [9]

Supplementary Table 2: Mechanisms linked with paravalvular leak in TAVR.

Authors	Year	Degree of patient specificity	Computational Approach	Main findings	Study scale/Sample	Validation approach
Zhang J. *et al* [10]	2025	patient-specific	FEA, validation against experimental results	Simulations showed that optimal balloon pressure balances flow area and TAVR-aortic root contact, while limiting deformation and PVL risk.	1 patient-specific aortic root model	experimental validation
Meng Z. *et al*], [11]	2024	patient-specific	FEA	A validated numerical framework simulating patient-specific valve deployment; showed implantation depth influences PVL and conduction block, supporting use of simulations to guide valve selection and release strategy.	6 patient-specific (1 BAV, and 5 tricuspid)	Compared with clinical/post-implantation outcomes and imaging
Spanjaards M. *et al*], [12]	2024	Idealised / synthetic	CFD, in vitro experiments, simplified mathematical model	Perform preoperative risk assessment of PVL using a simplified leakage model and study the effect of stent size and the degree of stenosis on the regurgitant volume.	2 synthetic average anatomies (female and male), with multiple stenosis / stent-size scenarios	In vitro validation, with comparison against CFD
Li. *et al* [13]	2023	patient-specific	FEA	Balloon post-dilation improves stent morphology and reduces PVL by over 30%, but increases aortic	2 patient-speicfic cases reconstructed from medical images	no

				stress by about 20% for self-expandable TAV devices.		
Dowling C. *et al* [14]	2022	patient-specific	FEA, CFD	Computer simulations predicting significant paravalvular regurgitation after TAVR identified patients with a higher risk of death within two years.	203 patients	Clinical outcome association (2-year mortality)
Prisco A.R. *et al* [15]	2022	patient-specific	CFD	PVL was found to decrease as the space occupying the PVL area increased, demonstrating that the native aortic valve contributes to reducing regurgitation.	1	Clinical/imaging consistency only; no formal external validation reported
Khodaei S. *et al*], [16]	2022	patient-specific	FSI + lumped-parameter model	PVL limits TAVR benefits by reducing maximum coronary flow rates by approximately 21% and increasing LV load by 17.57%, compared to TAVR with no PVL. PVL also decreases coronary wall shear stress, which may promote atherosclerosis development.	6 patients	Clinical Doppler echocardiography validation (velocity comparison in 2 sample patients; framework applied to 6 patients)
Finotello A. *et al* [17]	2021	Mixed *(device calibration from experiments + one patient-specific implantation simulation)*	FEA	Material properties of Nitinol TAV stents significantly impact simulated contact area, radial stresses imposed on host tissue, and PVL orifice area, highlighting the importance of device-specific	1 patient-specific simulation case, plus experimental radial force tests on 4 self-expanding devices	Experimental validation / device calibration

				material properties in TAVR modelling.		
Basri A.A. *et al* [18]	2021	patient-specific	FSI	PVL in TAVR increases blood velocity, pressure drop, and wall shear stress, leading to risks of recirculation flow, thrombus formation, and aortic wall damage.	1 patient-specific aorta model with simulated normal and PVL scenarios	no
Ghosh R.P. *et al* [19]	2020	Mixed	FEA, CFD, FSI	Optimal TAVR performance, minimizing complications like PVL, thrombogenicity, and valve migration, is achieved by deploying the valve at a specific depth in the ventricle.	Mixed setup: 1 patient-specific and common idealised model(s)	no
Spadaccio C. *et al* [20]	2020	patient-specific	FEA	FEA modelling enhanced CT predictions by demonstrating the impact of calcification on device misalignment and stent deformation, improving prediction of PVL occurrence.	1 patient case	No explicit external validation reported
Basri A.A. *et al* [21]	2020	patient-specific	FSI	Smaller valve openings in TAVR increase PVL likelihood, elevate recirculatory flow, and significantly impact hemodynamic parameters.	1 patient-specific case with 4 valve-opening scenarios (100%, 80%, 60%, 40% GOA)	Literature-based validation against prior published results
Luraghi G. *et al* [22]	2020	Idealised / synthetic	FSI	Different aortic root calcification patterns affect TAVR outcomes, with cusp coaptation calcifications causing mild PVL and	One idealised anatomical-resembling aortic root with multiple	No explicit external validation reported

				attachment line calcifications leading to moderate PVL in the anatomical-resembling aortic root model studied.	calcification-pattern scenarios	
Luraghi G. *et al* [23]	2019	patient-specific	FSI	Patient-specific FSI models accurately predict PVL with coherent regurgitant volume and orifice area showing a good qualitative and quantitative matching with postoperative CT scans and Doppler traces.	2 real clinical cases	Post-procedural CT and Doppler trace comparison
Bianchi M. *et al* [24]	2019	Patient-specific	FEA, CFD	Computational methods accurately predicted PVL and showed that optimizing TAV implantation depth and balloon inflation volume can significantly reduce PVL volume by up to 47%.	3 retrosepctive clinical cases affected by PVL	Post-procedural echocardiography comparison
Zhang G. *et al* [25]	2019	Patient-specific	FEA, ML	A combined FEA and machine learning model predicted aortic regurgitation with 90.9% accuracy by analysing the stress imposed on aortic tissue by the TAV, aiding in optimal valve size selection for TAVR patients.	22 patients	Internal model validation (using leave-one-out cross-validation)
Mao W. *et al* [26]	2018	patient-specific	FEA, CFD	Simulations show TAV orientation, skirt shape, and deployment height can significantly influence PVL	1 patient-specific aortic root model with multiple parametric	Echocardiography comparison

				severity, with variations in PVL volume up to 70%.	deployment scenarios	
Bosi, GM. *et al* [27]	2018	patient-specific	FEA	FEA models showed a mean difference of 2.5% between predicted and actual stent diameters and detected PVL in 79% of cases in balloon-expandable Sapien XT devices, aiding in accurate pre-procedural planning for TAVR.	14 patient-specific models	Post-procedural CT diameter comparison / imaging-based validation
Basri A.A. *et al* [28]	2016	patient-specific	FSI	Simulation of undersized TAVR valve leads to PVL, causing significant aortic wall deformation, aortic blood flow recirculation, and increased risks of serious complications.	1 patient-specific aorta model	Literature-based validation against prior published results
Bianchi M. . *et al* [29]	2015	Mixed	FEA	Deployment simulations show the role that calcifications deposits may play in sub-optimal valve anchoring of TAVR to the aortic root wall, leading to the presence of gaps that result in PVL.	Patient-specific deployment models with 3 axial deployment positions; separate crimping comparison of 2 valve designs (Edwards SAPIEN and Polynova)	No explicit external validation clearly reported, although experimental material characterisation was used for model inputs
Morganti, S. *et al* [30]	2014	patient-specific	FEA	Use FEA to study the impact of stent apposition, and aortic root anatomy on PVL, showing good agreement with post-operative clinical data.	2 real clinical cases	Post-procedural imaging comparison

Modelling studies addressing PVL summarised in Supplementary Table 2: [10], [11], [12], [13], [14], [15], [16], [17], [18], [19], [20], [21], [22], [23], [24], [25], [26], [27], [28], [29], [30]

Supplementary Table 3: Impact on coronary flow and risk of coronary obstruction with TAVR.

Authors	Year	Degree of patient specificity	Computational Approach	Main findings	Study scale/Sample	Validation approach
Fan J. *et al* [31]	2024	Patient-specific	FEA	Patient-specific simulations accurately predicted frame deformation and showed decreasing coronary ostia–closest device structure distance with higher obstruction risk, linking predicted distance to CO risk.	14 patient-specific anatomies with patients' calcified native valve leaflets, with 1 TAV device as implanted in the patient	Simulated device deformation compared with post-TAVR CT images of clinically implanted device. Simulated metrics compared with clinical outcomes to identify patterns.
Oks D. *et al* [32]	2023	Idealised	FSI	Commissural misalignment in simulated TAVR reduced coronary perfusion by 3.2% and single coronary branch flow by 6.8%, increased systolic transvalvular pressure gradients by 5.3%, and diastolic leaflet stresses by 16.0%, highlighting the need for improved alignment control in TAVR systems.	1 idealised aortic anatomy, with 1 TAV device	No explicit external validation reported
Scuoppo R. *et al* [33]	2023	Patient-specific	FEA	High implantation depth and undersizing the second transcatheter heart valve in a simulated TAVR-in-TAVR deployment significantly reduced coronary flow to 20% of pre-TAVR levels. A positive	1 patient-specific anatomy with parameterised model of native leaflets and patient's calcifications, with 1 valve-in-valve TAVR device and 4	Comparison of simulated TAVR deformation with clinically observed deformation on post-TAVR CT

				correlation between coronary flow and valve-to-coronary distance was observed, highlighting the importance of these factors in pre-procedural planning of TAVR-in-TAVR.	deployment depth scenarios	
Heitkemper M. *et al* [34]	2020	Patient-specific	FEA, *in vitro* experiments	The distance from cusp to coronary ostium indexed by coronary artery diameter (DLC/d), evaluated from FEA simulations, provides a more predictive measure of coronary obstruction risk than traditional measures like coronary ostium height and sinus of Valsalva diameter, with a cutoff of DLC/d < 0.7 yielding 100% sensitivity and 95.7% specificity.	28 patient-specific anatomies including native leaflets and calcium deposits, with 1 idealised TAVR device represented by an expandable cylinder	Comparison of simulated metrics against in vitro experiments of TAVR implantation in flexible, 3D printed patient-specific anatomies.
Wald S. *et al* [35]	2018	Idealised	FSI	FSI models confirm the paradox of increased rest coronary blood flow in AS and its reduction post-TAVR via valve leaflet dynamics.	1 idealised aortic anatomy with 1 healthy, 2 stenotic, and 2 types of TAVR valve scenarios	Brief comparison with parallel in vitro study conducted of three of the considered scenarios.
Kandail H.S. *et al* [36]	2018	Mixed *(patient-specific anatomy, idealised boundary conditions)*	FSI	Simulated supra-annular CoreValve deployment results in higher para-valvular flow to coronary arteries, and significantly elevated wall shear stress (WSS) in coronary arteries and aorta compared to annular deployment.	1 patient-specific anatomy (from post-TAVR CT), with 1 TAVR device at 2 implantation depths	None

Modelling studies investigating the risk of coronary obstruction summarised in Supplementary Table 3: [31], [32], [33], [34], [35], [36]

Supplementary Table 4: Factors involved in cardiac conduction abnormalities post-TAVR.

Authors	Year	Degree of patient specificity	Computational Approach	Main findings	Study scale/Sample	Validation approach
Reza S. *et al* [37]	2022	Patient-specific	FEA	FEA simulations show elevated stresses in the membranous septum region correlated with CCA risk post-TAVR, with area-weighted average maximum principal logarithmic strain emerging as a strong predictor for CCA risk that could be used to minimize CCA risk through preprocedural planning.	2 patient-specific anatomies including leaflets and calcium deposits, with the 1 TAVR device implanted in the patient at 3 depths (1 matching patients' post-TAVR CT)	Simulated metrics compared with clinical outcomes to identify patterns.
Bosi G.M. *et al* [38]	2020	Patient-specific	FEA	FEA model shows good agreement with clinical outcomes, accurately predicting PVL in 83% of cases and identifying high strains associated with the need for a pacemaker.	28 patient-specific aortic anatomies including native leaflets and calcium deposits, each with 1 TAVR device implanted virtually in line with clinical guidelines	Simulated metrics compared against post-TAVR clinical fluroscopy and echocardiography, as well as clinical outcomes.
McGee OM. *et al* [39]	2019	Patient-specific	FEA	Implantation depth significantly influences stresses in the aortic sinus and TAV frame eccentricity. Sub-annular positioning reduces peak aortic sinus stress but increases stress near the conductance system, while supra-	1 patient-specific anatomy including leaflets and calcium deposits, with 1 TAVR device implanted at 3 deployment depths.	None

				annular positioning has the opposite effect.		

Modelling studies focused on CCA summarised in Supplementary Table 4: [37], [38], [39]

Supplementary Table 5: Other procedural TAVR complications.

Authors	Year	Degree of patient specificity	Computational Approach	Main findings	Study scale/Sample	Validation approach
Wen J. *et al* [40]	2023	Mixed *(patient-specific anatomy of healthy patients adjusted to represent TAVR patients, standard inflow waveform)*	CFD	Elliptical annulus shapes after TAVR (uneven valve expansion) significantly disturb haemodynamics in the ascending aorta, with regions of low time-averaged wall shear stress (TAWSS) alongside elevated oscillatory shear index (OSI) and cross-flow index (CFI, suggesting a mechanism for adverse aortic loading post-TAVR.	8 patient-specific aortic anatomies (but of healthy patients), manipulated to generate elliptic annular geometry representative of post-TAVR conditions	None
Ibanez I. *et al* [41]	2021	Mixed *(patient-specific anatomy and echo-derived simplified valve representation, inflow waveform from literature)*	FSI	A 4° inclination of the TAVR valve towards the left main coronary artery reduces high wall shear stress and pressure in the ascending aorta, potentially mitigating adverse hemodynamic effects and improving the longevity of the prosthesis.	1 patient-specific anatomy, with simplified representation of 1 TAVR device at 4 different inclination angles	None
Caballero A. *et al* [42]	2020	Patient-specific	FEA, FSI	Comparative simulations show balloon-expandable (BE) TAVR deployment at an optimal height to give	1 patient-specific anatomy, with TAVR procedure virtually simulated	Comparison of simulated flow with clinically measured echocardiography metrics in previous study of

				the lowest regurgitant volume, while self-expanding (SE) TAVR deployment had higher regurgitant volumes. High deployment of BE devices also provided the highest MR reduction and lowest stress on native leaflets.	using a SE valve at three implantation height	different TAVR device [44], no validation of the findings in this study.
Wu M.C.H. *et al* [43]	2019	Idealised	FSI	The ratio of radial outward force to friction force between aortic wall and device can predict the likelihood of TAV migration, providing insights for improving anchoring strategies in TAVR.	1 idealised aortic geometry based on average adult aorta sizes, with 1 TAVR device	Methodology for TAVR stent representation validated against benchmark bending beam problem and experimental tension testing. No validation of full simulations.
Caballero A. *et al* [44]	2019	Patient-specific	FEA, FSI	Simulation of three different TAVR implantation heights of a balloon-expandable device affects aortic-mitral coupling and mitral regurgitaion severity, with the midway implantation model providing the best hemodynamic performance and MR reduction in the simulated anatomy (bicuspid aortic valve).	1 patient-specific anatomy including aortic root, native valve and calcification, and mitral valve, chordae, myocardium and left ventricle/atrial endocardial walls, with 1 TAVR device at 3 implantation depths.	Comparison of simulated flow with clinically measured echocardiography in pre-TAVR scenario, and claim agreement of all three post-TAVR scenarios with clinical post-TAVR echocardiography. Clinically important echo metrics showed poorer agreement (e.g. max AV velocity and mean gradient)

Conti M. *et al* [45]	2019	Idealised	CFD, *in-vitro* experiments	In vitro experiments and CFD analysis in an idealised aortic anatomy show that a constant retrograde auxiliary flow of 0.5 L/min from the right subclavian artery could protect the brachiocephalic trunk from particle embolisms during TAVR.	1 idealised aorta resembling average healthy anatomy	Comparison with in vitro experiment replicating simulated problem.
Ishii, M. *et al* [46]	2019	Patient-specific	CFD, *in vitro* experiments (micro-fluidic blood analysis)	CFD, in combination with experimental analysis using the Total Thrombus-formation Analysis System (T-TAS), a microchip-based flow chamber system to assess whole blood thrombogenicity, shows significant reduction in aortic wall shear stress and total thrombogenicity post-TAVR, respectively, with thrombogenicity increasing gradually over 30 days, helping to predict bleeding risk post-TAVR.	5 patient-specific anatomies including native leaflets	None for CFD element of study.
Tan F.P.P. *et al* [47]	2012	Patient-specific	CFD	Show restored normal aortic flow with uniform WSS and reduced turbulence post-TAVR compared to pre-TAVR using MRI patient data.	1 patient-specific aortic anatomy (above the aortic root)	None

Modelling studies addressing less common procedural complications summarised in Supplementary Table 5: [40], [41], [42], [43], [44], [45], [46], [47]

Supplementary Table 6: Planning TAVR in challenging scenarios.

Authors	Year	Degree of patient specificity	Computational Approach	Main findings	Study scale/Sample	Validation approach
Bicuspid aortic valve						
An K. *et al* [48]	2024	Patient-specific	CFD	In bicuspid aortic valve with ascending aortic dilation, CFD showed abnormal vortical/helical flow and elevated wall shear stress (WSS) in the ascending aorta pre-TAVR, which improved with reduced WSS post-TAVR, suggesting TAVR may not exacerbate aortic dilation, though long-term follow-up is needed	3 patients pre- and post-TAVR, each with 1 TAVR device stent segmented from post-TAVR imaging	None
Zhang X. *et al* [49]	2024	Patient-specific	FEA	Computational models identified three stages of balloon burst, with high stress at folding edges due to calcifications causing transverse bursts, and sharp calcifications leading to rapid crack propagation and pinhole ruptures, guiding better balloon selection and sizing in TAVR for bicuspid aortic valve.	2 patient-specific anatomies including calcification and parametric model of native leaflets, with 1 TAVR balloon configuration and different material parameters of calcifications and balloon	Comparison of non-constrained behaviour against manufacturer data, comparison of simulated balloon burst with clinical procedural observations

Helbock RT. *et al* [50]	2023	Patient-specific	FEA, CFD	Simulation of the first-eccentric dedicated TAV design in bicuspid aortic valve. showed improved hemodynamic performance with lower jet velocity and wall shear stress compared to commercial TAVR valves.	6 patient-specific anatomies with native leaflets and calcium deposits, with 2 TAVR devices under different deployment and flow conditions	None
Wei H. *et al* [51]	2023	Idealised	CFD	TAV deployment height and orientation significantly affect coronary haemodynamics, with certain configurations improving coronary perfusion pressure by up to 83% compared to pre-deployment levels.	3 idealised bicuspid aortic valve type models based on physiology, with 1 TAVR device deployed at 3 heights and 2 orientrations	None
Anam SB. *et al* [52]	2022	Patient-specific	FEA, CFD	Newer generation TAV devices significantly reduce PVL and thrombogenic risk compared to older devices in bicuspid aortic valve patients.	3 patient-specific models with parameterised native leaflets and patient-specific calcium deposits, each with 1 TAVR device actually implanted in the patient and 1 new generation TAVR device	Comparison with echocardiography and severity grading of clinically observed paravalvular leak post-TAVR for clinically implanted TAVR device
Anam SB. *et al* [53]	2022	Mixed *(patient-specific geometry, boundary*	CFD, *in vitro* experiments	Model TAVR device deployment in both computational and physical models, to assess degrees	3 patient-specific models with parameterised native leaflets and patient-	Comparison with analagous in vitro experiments using 3D printed patient-specific anatomies, and comparison

		conditions taken from analagous in vitro experiment with standard flow conditions)		of PVL, demonstrating good agreement with clinical data.	specific calcium deposits, with 1 TAVR device actually implanted in each patient at clinically deployed depth	with echocardiography and severity grading of clinically observed paravalvular leak post-TAVR for clinically implanted TAVR device
Finotello A. *et al* [54]	2021	Patient-specific	FEA	In four BAV patients, TAV devices with high radial force produced lower paravalvular orifice area and higher stent-root contact area than high-conformability devices, with the authors concluding that high radial force may be a more desirable characteristic for TAVR in bicuspid aortic valve anatomy.	4 patient-specific anatomies including parametric reconstruction of native leaflets and patient-specific calcifications	Qualitative comparison of deployed position and deformation and quantitative comparison of device diameter with procedural fluoroscopy images from clinically implanted device. No validation of PVL findings.
Dowling C. *et al* [55]	2021	Mixed *(patient-specific geometry, standard pressure difference used for CFD simulation)*	FEA, CFD	Optimal TAV sizing and positioning improves outcomes in bicuspid aortic valve patients, reducing PVL and mortality.	50 patient-specific anatomies including the native aortic leaflets and calcifications, each with 2 TAVR devices, each at 2 implant depths	Comparison of clinical outcomes and post-TAVR echocardiography assessment of PVL between closest simulated scenario and clinically observed TAVR implantation.
Liu X. *et al* [56]	2021	Patient-specific	FEA	Simulations show more TAVR skirt malapposition in bicuspid vs tricuspid aortic valve, correlating with PVL severity and	43 patient-specific anatomies including calcified native leaflets, each with 1 TAVR device actually	Visual and quantitative comparison of simulated implantation with post-procedural CT imaging of implanted device; simulated

				mainly driven by malapposition at the interleaflet triangles.	implanted clinically at clinically achieved implantation depth	malposition compared with post-procedural echocardiography assessment of PVL; comparison and calibration of simulated TAVR stent model against experimental radial force test.
Pasta S. *et al* [57]	2020	Mixed *(patient-specific geometry, standard physiological waveform boundary condition)*	FSI	Edwards Sapien 3 valves expand well in bicuspid aortic valve anatomy, with accurate predictions of stent deformity and PVL flows.	9 patient-specific anatomies including parameteric model of native valve leaflets and patient-specific calcifications, each with 1 TAVR device actually implanted clinically at clinically achieved implantation depth	Comparison of simulated deformity and eccentricity against post-TAVR CT imaging of implanted device; comparison of simulated PVL flow against PVL assessed by post-TAVR echocardiography.
Pasta S. *et al* [58]	2021	Mixed *(patient-specific geometry, standard physiological waveform boundary condition)*	FEA, FSI	Simulations show slight asymmetric and elliptical expansion of self-expanding Evolut Pro stent frame in bicuspid aortic valve anatomy, and identified high-risk regions for PVL.	6 patient-specific anatomies including parametric model of native leaflets and patient-specific calcifications, and 1 TAVR device	None
Lavon K. *et al* [59]	2019	Mixed *(patient-specific geometry, standard pressure difference used*	FEA, CFD	Aligning TAV commissures with native commissures minimized PVL, and Evolut PRO device reduced PVL by half compared to the Evolut	1 patient-specific anatomy including parametric model of native leaflets and patient-specific calcifications, with 2	None

		for CFD simulation)		R in bicuspid aortic valve patients.	TAVR devices, each at 5 TAV leaflet orientations and 3 deployment depths	
Dowling C. *et al* [60]	2019	Mixed *(patient-specific geometry, standard pressure difference used for CFD simulation)*	FEA, CFD	Patient-specific simulations accurately predict TAV deformation, PVL, and conduction disturbances, and optimal TAV sizing and positioning improves TAVR outcomes in bicuspid aortic valve patients.	37 patient-specific anatomies including native leaflets and calcifications, with 1 TAVR device at clinically implanted depth	Comparison of implantation with post-procedural CT imaging of implanted TAVR; comparison of simulated PVL with clinically observed PVL on post-TAVR echocardiography; comparison of simulated metrics with clinical outcomes; comparison and calibration of stent model with experimental radial force testing
Other complex scenarios						
Khodaei S. *et al* [61]	2021	Patient-specific	CFD, FSI	Patient-specific framework for C3VD (complex valvular, ventricular and vascular disease) patients undergoing TAVR provides accurate blood flow metrics and novel diagnostic insights, validated against clinical cardiac catheterization and Doppler echocardiographic measurements.	11 patient-specific anatomies of the left ventricle post-TAVR with reduced order model of TAVR valve, using patient-specific inflow and outflow boundary conditions and material properties calibrated from echocardiography data	Comparison with clinical cardiac catheterization and Doppler echocardiographic images and measurements

Khodaei S. *et al* [62]	2021	Patient-specific	CFD, FSI	Develop a patient-specific diagnostic framework to quantify global and local haemodynamics for C3VD (complex valvular, ventricular and vascular disease) patients undergoing TAVR, validated against clinical data.	3 patient-specific anatomy of the post-TAVR left ventricle with reduced order model of TAVR valve, using patient-specific inflow and outflow boundary conditions and material properties calibrated from echocardiography data	Comparison with clinical cardiac catheterization and Doppler echocardiographic images and measurements
Capelli C. *et al* [63]	2012	Patient-specific	FEA	Use patient-specific data to explore the feasibility of TAVR in morphologies, such as Valve-in-Valve and aortic regurgitation, which are currently borderline cases for a percutaneous approach.	5 patient-specific anatomies including native leaflets and existing bioprostheses, with 1 TAVR device across all cases	None
Dwyer H.A. *et al* [64]	2009	Idealised	CFD	Use computational models to assess the feasibility of TAVR in aortic regurgitation, by assessing the total force on the device that can potentially lead to its migration.	1 idealised aortic anatomy with aortic root dimensions derived from the literature, with 1 idealised TAVR device	None
Sirset-Becker T. *et al* [65]	2025	Patient-specific	FEA	Computational simulations showed ViV was generally feasible after aortic root replacement followed by implantation of a composite valve graft.	40 patient-specific anatomies including previous bioprosthetic replacement valve and calcifications, each with 2 TAVR devices at	Visual and quantitative comparison of simulated TAVR implantation with post-TAVR CT imaging in 2 additional patient-specific

				However, balloon-expandable valves carried coronary obstruction risk and self-expanding valves risked frame deformation at the graft anastomosis	fixed implant depths + 2 additional patient-specific anatomies used for validation	cases to test accuracy of modelling approach

Relevant studies addressing computational modelling in complex TAVR scenarios summarised in Supplementary Table 6: [48], [49], [50], [51], [52], [53], [54], [55], [56], [57], [58], [59], [60], [61], [62], [63], [64], [65]

Supplementary References

[1] D. Carbonaro *et al.*, "Impact of nickel–titanium super-elastic material properties on the mechanical performance of self-expandable transcatheter aortic valves," *J. Mech. Behav. Biomed. Mater.*, vol. 138, Feb. 2023, doi: 10.1016/j.jmbbm.2022.105623.

[2] D. Carbonaro, D. Gallo, U. Morbiducci, A. Audenino, and C. Chiastra, "In silico biomechanical design of the metal frame of transcatheter aortic valves: multi-objective shape and cross-sectional size optimization," Structural and Multidisciplinary Optimization, vol. 64, no. 4, pp. 1825–1842, 2021, doi: 10.1007/s00158-021-02944-w.

[3] M. Gessat *et al.*, "Image-based mechanical analysis of stent deformation: Concept and exemplary implementation for aortic valve stents," *IEEE Trans. Biomed. Eng.*, vol. 61, no. 1, pp. 4–15, Jan. 2014, doi: 10.1109/TBME.2013.2273496.

[4] O. Mutlu *et al.*, "Finite Element Analysis of Evolut Transcatheter Heart Valves: Effects of Aortic Geometries and Valve Sizes on Post-TAVI Wall Stresses and Deformations," *J. Clin. Med.*, vol. 14, no. 3, Feb. 2025, doi: 10.3390/JCM14030850.

[5] A. Morany, R. G. Bardon, K. Lavon, A. Hamdan, D. Bluestein, and R. Haj-Ali, "Analysis of fibrocalcific aortic valve stenosis: computational pre-and-post TAVR haemodynamics behaviours," *R. Soc. Open Sci.*, vol. 11, no. 2, Feb. 2024, doi: 10.1098/RSOS.230905.

[6] J. Li, W. Yan, W. Wang, S. Wang, and L. Wei, "Comparison of Balloon-Expandable Valve and Self-Expandable Valve in Transcatheter Aortic Valve Replacement: A Patient-Specific Numerical Study," *J. Biomech. Eng.*, vol. 144, no. 10, Oct. 2022, doi: 10.1115/1.4054332.

[7] V. Govindarajan, A. Kolanjiyil, N. P. Johnson, H. Kim, K. B. Chandran, and D. D. McPherson, "Improving transcatheter aortic valve interventional predictability via fluid-structure interaction modelling using patient-specific anatomy," *R. Soc. Open Sci.*, vol. 9, no. 2, 2022, doi: 10.1098/rsos.211694.

[8] M. Bongert *et al.*, "Comparison of two biological aortic valve prostheses inside patient-specific aorta model by bi-directional fluid-structure interaction," in *Current Directions in Biomedical Engineering*, Walter de Gruyter GmbH, Sep. 2018, pp. 59–62. doi: 10.1515/cdbme-2018-0015.

[9] C. Russ *et al.*, "Simulation of transcatheter aortic valve implantation under consideration of leaflet calcification," in *2013 35th Annual International Conference of the IEEE Engineering in Medicine and Biology Society (EMBC)*, Jul. 2013, pp. 711–714. doi: 10.1109/EMBC.2013.6609599.

[10] J. Zhang *et al.*, "Computational evaluation of interactive dynamics for a full transcatheter aortic valve device in a patient-specific aortic root," *Comput. Biol. Med.*, vol. 185, Feb. 2025, doi: 10.1016/j.compbiomed.2024.109512.

[11] Z. Meng *et al.*, "Computational study of transcatheter aortic valve replacement based on patient-specific models-rapid surgical planning for self-expanding valves," *Front. Physiol.*, vol. 15, 2024, doi: 10.3389/FPHYS.2024.1407215.

[12] M. Spanjaards, F. Borowski, L. Supp, R. Ubachs, V. Lavezzo, and O. van der Sluis, "A fast in silico model for preoperative risk assessment of paravalvular leakage," *Biomech. Model. Mechanobiol.*, 2024, doi: 10.1007/s10237-024-01816-8.

[13] J. Li, Z. Meng, W. Yan, W. Wang, L. Wei, and S. Wang, "Computational study of the balloon dilation steps on transcatheter aortic valve replacement," *Front. Bioeng. Biotechnol.*, vol. 11, 2023, doi: 10.3389/fbioe.2023.1333138.

[14] C. Dowling, R. Gooley, L. McCormick, S. Firoozi, and S. J. Brecker, "Patient-specific computer simulation to predict long-term outcomes after transcatheter aortic valve replacement," *J. Cardiovasc. Comput. Tomogr.*, vol. 16, no. 3, pp. 254–261, 2022, doi: 10.1016/j.jcct.2021.11.014.

[15] A. R. Prisco *et al.*, "The native aortic valve reduces paravalvular leak in TAVR patients," *Front. Physiol.*, vol. 13, 2022, doi: 10.3389/fphys.2022.910016.

[16] S. Khodaei, L. Garber, J. Bauer, A. Emadi, and Z. Keshavarz-Motamed, “Long-term prognostic impact of paravalvular leakage on coronary artery disease requires patient-specific quantification of hemodynamics,” *Sci. Rep.*, vol. 12, no. 1, Dec. 2022, doi: 10.1038/s41598-022-21104-8.

[17] A. Finotello, R. Gorla, N. Brambilla, F. Bedogni, F. Auricchio, and S. Morganti, “Finite element analysis of transcatheter aortic valve implantation: Insights on the modelling of self-expandable devices,” *J. Mech. Behav. Biomed. Mater.*, vol. 123, Nov. 2021, doi: 10.1016/j.jmbbm.2021.104772.

[18] A. A. Basri *et al.*, “Fluid-Structure Interaction in Problems of Patient Specific Transcatheter Aortic Valve Implantation with and Without Paravalvular Leakage Complication,” *Fluid Dynamics and Materials Processing*, vol. 17, no. 3, pp. 531–553, 2021, doi: 10.32604/fdmp.2021.010925.

[19] R. P. Ghosh, G. Marom, M. Bianchi, K. D’souza, W. Zietak, and D. Bluestein, “Numerical evaluation of transcatheter aortic valve performance during heart beating and its post-deployment fluid–structure interaction analysis,” *Biomech. Model. Mechanobiol.*, vol. 19, no. 5, pp. 1725–1740, 2020, doi: 10.1007/s10237-020-01304-9.

[20] C. Spadaccio *et al.*, “Bioengineering case study to evaluate complications of adverse anatomy of aortic root in transcatheter aortic valve replacement: Combining biomechanical modelling with CT imaging,” *Bioengineering*, vol. 7, no. 4, pp. 1–10, 2020, doi: 10.3390/bioengineering7040121.

[21] A. A. Basri *et al.*, “Fluid Structure Interaction on Paravalvular Leakage of Transcatheter Aortic Valve Implantation Related to Aortic Stenosis: A Patient-Specific Case,” *Comput. Math. Methods Med.*, vol. 2020, 2020, doi: 10.1155/2020/9163085.

[22] G. Luraghi, J. F. R. Matas, M. Beretta, N. Chiozzi, L. Iannetti, and F. Migliavacca, “The impact of calcification patterns in transcatheter aortic valve performance: a fluid-structure interaction analysis,” *Comput. Methods Biomech. Biomed. Engin.*, vol. 24, no. 4, pp. 375–383, 2020, doi: 10.1080/10255842.2020.1817409.

[23] G. Luraghi *et al.*, “On the Modeling of Patient-Specific Transcatheter Aortic Valve Replacement: A Fluid–Structure Interaction Approach,” *Cardiovasc. Eng. Technol.*, vol. 10, no. 3, pp. 437–455, 2019, doi: 10.1007/s13239-019-00427-0.

[24] M. Bianchi *et al.*, “Patient-specific simulation of transcatheter aortic valve replacement: impact of deployment options on paravalvular leakage,” *Biomech. Model. Mechanobiol.*, vol. 18, no. 2, pp. 435–451, 2019, doi: 10.1007/s10237-018-1094-8.

[25] G. Zhang, M. Pu, Y. Gu, and X. Zhou, “Predicting aortic regurgitation after transcatheter aortic valve replacement by finite element method,” *IEEE Access*, vol. 7, pp. 64315–64322, 2019, doi: 10.1109/ACCESS.2019.2916762.

[26] W. Mao, Q. Wang, S. Kodali, and W. Sun, “Numerical Parametric Study of Paravalvular Leak Following a Transcatheter Aortic Valve Deployment into a Patient-Specific Aortic Root,” *J. Biomech. Eng.*, vol. 140, no. 10, 2018, doi: 10.1115/1.4040457.

[27] G. M. Bosi *et al.*, “Population-specific material properties of the implantation site for transcatheter aortic valve replacement finite element simulations,” *J. Biomech.*, vol. 71, pp. 236–244, Apr. 2018, doi: 10.1016/j.jbiomech.2018.02.017.

[28] A. A. Basri *et al.*, “The hemodynamic effects of paravalvular leakage using fluid structure interaction; Transcatheter aortic valve implantation patient,” *J. Med. Imaging Health Inform.*, vol. 6, no. 6, pp. 1513–1518, 2016, doi: 10.1166/jmihi.2016.1840.

[29] M. Bianchi, R. P. Ghosh, G. Marom, M. J. Slepian, and D. Bluestein, “Simulation of Transcatheter Aortic Valve Replacement in patient-specific aortic roots: Effect of crimping and positioning on device performance,” *Annu. Int. Conf. IEEE Eng. Med. Biol. Soc.*, vol. 2015, pp. 282–285, Nov. 2015, doi: 10.1109/EMBC.2015.7318355.

[30] S. Morganti *et al.*, "Simulation of transcatheter aortic valve implantation through patient-specific finite element analysis: Two clinical cases," *J. Biomech.*, vol. 47, no. 11, pp. 2547–2555, Aug. 2014, doi: 10.1016/j.jbiomech.2014.06.007.
[31] J. Fan *et al.*, "Coronary obstruction analysis in transcatheter aortic valve implantation through patient-specific computational modelling," *Front. Cardiovasc. Med.*, vol. 11, 2024, doi: 10.3389/FCVM.2024.1432235.
[32] D. Oks, G. Houzeaux, M. Vázquez, M. Neidlin, and C. Samaniego, "Effect of TAVR commissural alignment on coronary flow: A fluid-structure interaction analysis," *Comput. Methods Programs Biomed.*, vol. 242, Dec. 2023, doi: 10.1016/j.cmpb.2023.107818.
[33] R. Scuoppo, S. Cannata, G. Gentile, C. Gandolfo, and S. Pasta, "Parametric analysis of transcatheter aortic valve replacement in transcatheter aortic valve replacement: evaluation of coronary flow obstruction," *Front. Bioeng. Biotechnol.*, vol. 11, 2023, doi: 10.3389/fbioe.2023.1267986.
[34] M. Heitkemper *et al.*, "Modeling risk of coronary obstruction during transcatheter aortic valve replacement," *Journal of Thoracic and Cardiovascular Surgery*, vol. 159, no. 3, pp. 829-838.e3, 2020, doi: 10.1016/j.jtcvs.2019.04.091.
[35] S. Wald, A. Liberzon, and I. Avrahami, "A numerical study of the hemodynamic effect of the aortic valve on coronary flow," *Biomech. Model. Mechanobiol.*, vol. 17, no. 2, pp. 319–338, Apr. 2018, doi: 10.1007/s10237-017-0962-y.
[36] H. S. Kandail *et al.*, "Impact of annular and supra-annular CoreValve deployment locations on aortic and coronary artery hemodynamics," *J. Mech. Behav. Biomed. Mater.*, vol. 86, pp. 131–142, 2018, doi: 10.1016/j.jmbbm.2018.06.032.
[37] S. Reza *et al.*, "A computational framework for post-TAVR cardiac conduction abnormality (CCA) risk assessment in patient-specific anatomy," *Artif. Organs*, vol. 46, no. 7, pp. 1305–1317, Jul. 2022, doi: 10.1111/aor.14189.
[38] G. M. Bosi *et al.*, "A validated computational framework to predict outcomes in TAVI," *Sci. Rep.*, vol. 10, no. 1, 2020, doi: 10.1038/s41598-020-66899-6.
[39] O. M. McGee, P. S. Gunning, A. McNamara, and L. M. McNamara, "The impact of implantation depth of the Lotus™ valve on mechanical stress in close proximity to the bundle of His," *Biomech. Model. Mechanobiol.*, vol. 18, no. 1, pp. 79–88, Feb. 2019, doi: 10.1007/s10237-018-1069-9.
[40] J. Wen *et al.*, "Risk evaluation of adverse aortic events in patients with non-circular aortic annulus after transcatheter aortic valve implantation: a numerical study," *Biomech. Model. Mechanobiol.*, vol. 22, no. 4, pp. 1379–1394, Aug. 2023, doi: 10.1007/s10237-023-01725-2.
[41] I. Ibanez, B. A. de Azevedo Gomes, and A. O. Nieckele, "Effect of percutaneous aortic valve position on stress map in ascending aorta: A fluid-structure interaction analysis," *Artif. Organs*, vol. 45, no. 7, pp. O195–O206, 2021, doi: 10.1111/aor.13883.
[42] A. Caballero, W. Mao, R. McKay, and W. Sun, "The Impact of Self-Expandable Transcatheter Aortic Valve Replacement on Concomitant Functional Mitral Regurgitation: A Comprehensive Engineering Analysis," *Structural Heart*, vol. 4, no. 3, pp. 179–191, May 2020, doi: 10.1080/24748706.2020.1740365.
[43] M. C. H. Wu, H. M. Muchowski, E. L. Johnson, M. R. Rajanna, and M. C. Hsu, "Immersogeometric fluid–structure interaction modeling and simulation of transcatheter aortic valve replacement," *Comput. Methods Appl. Mech. Eng.*, vol. 357, Dec. 2019, doi: 10.1016/j.cma.2019.07.025.
[44] A. Caballero, W. Mao, R. McKay, and W. Sun, "The impact of balloon-expandable transcatheter aortic valve replacement on concomitant mitral regurgitation: A comprehensive computational analysis," *J. R. Soc. Interface*, vol. 16, no. 157, 2019, doi: 10.1098/rsif.2019.0355.

[45] M. Conti *et al.*, “Reversed Auxiliary Flow to Reduce Embolism Risk During TAVI: A Computational Simulation and Experimental Study,” *Cardiovasc. Eng. Technol.*, vol. 10, no. 1, pp. 124–135, 2019, doi: 10.1007/s13239-018-00386-y.
[46] M. Ishii *et al.*, “Reduction in thrombogenic activity and thrombocytopenia after transcatheter aortic valve implantation — The ATTRACTIVE-TTAS study,” *IJC Heart and Vasculature*, vol. 23, 2019, doi: 10.1016/j.ijcha.2019.100346.
[47] F. P. P. Tan *et al.*, “Comparison of Aortic Flow Patterns Before and After Transcatheter Aortic Valve Implantation,” *Cardiovasc. Eng. Technol.*, vol. 3, no. 1, pp. 123–135, 2012, doi: 10.1007/s13239-011-0073-3.
[48] K. An, F. Zhang, W. Ouyang, and X. Pan, “Blood flow dynamics in the ascending aorta of patients with bicuspid aortic valve before and after transcatheter aortic valve replacement: a computational fluid dynamics study,” *BMC Cardiovasc. Disord.*, vol. 24, no. 1, Dec. 2024, doi: 10.1186/S12872-024-04394-W.
[49] X. Zhang *et al.*, “Mechanism of balloon burst during transcatheter aortic valve replacement pre-dilatation: Image observation and validation by finite element analysis,” *Comput. Biol. Med.*, vol. 168, Jan. 2024, doi: 10.1016/j.compbiomed.2023.107714.
[50] R. T. Helbock *et al.*, “Designing a Novel Asymmetric Transcatheter Aortic Valve for Stenotic Bicuspid Aortic Valves Using Patient-Specific Computational Modeling,” *Ann. Biomed. Eng.*, vol. 51, no. 1, pp. 58–70, Jan. 2023, doi: 10.1007/s10439-022-03039-3.
[51] H. Wei *et al.*, “Effect of transcatheter aortic valve replacement on bicuspid coronary hemodynamics: A numerical study,” *Med. Nov. Technol. Devices*, vol. 18, Jun. 2023, doi: 10.1016/j.medntd.2023.100239.
[52] S. B. Anam *et al.*, “Assessment of Paravalvular Leak Severity and Thrombogenic Potential in Transcatheter Bicuspid Aortic Valve Replacements Using Patient-Specific Computational Modeling,” *J. Cardiovasc. Transl. Res.*, vol. 15, no. 4, pp. 834–844, Aug. 2022, doi: 10.1007/s12265-021-10191-z.
[53] S. B. Anam *et al.*, “Validating In Silico and In Vitro Patient-Specific Structural and Flow Models with Transcatheter Bicuspid Aortic Valve Replacement Procedure,” *Cardiovasc. Eng. Technol.*, vol. 13, no. 6, pp. 840–856, 2022, doi: 10.1007/s13239-022-00620-8.
[54] A. Finotello *et al.*, “Performance of high conformability vs. high radial force devices in the virtual treatment of TAVI patients with bicuspid aortic valve,” *Med. Eng. Phys.*, vol. 89, pp. 42–50, Mar. 2021, doi: 10.1016/j.medengphy.2021.02.004.
[55] C. Dowling *et al.*, “Patient-Specific Computer Simulation to Optimize Transcatheter Heart Valve Sizing and Positioning in Bicuspid Aortic Valve,” *Structural Heart*, vol. 5, no. 6, pp. 621–630, 2021, doi: 10.1080/24748706.2021.1991604.
[56] X. Liu *et al.*, “Sealing Behavior in Transcatheter Bicuspid and Tricuspid Aortic Valves Replacement Through Patient-Specific Computational Modeling,” *Front. Cardiovasc. Med.*, vol. 8, 2021, doi: 10.3389/fcvm.2021.732784.
[57] S. Pasta *et al.*, “Simulation study of transcatheter heart valve implantation in patients with stenotic bicuspid aortic valve,” *Med. Biol. Eng. Comput.*, vol. 58, no. 4, pp. 815–829, Apr. 2020, doi: 10.1007/s11517-020-02138-4.
[58] S. Pasta *et al.*, “Transcatheter heart valve implantation in bicuspid patients with self-expanding device,” *Bioengineering*, vol. 8, no. 7, 2021, doi: 10.3390/bioengineering8070091.
[59] K. Lavon *et al.*, “Biomechanical modeling of transcatheter aortic valve replacement in a stenotic bicuspid aortic valve: deployments and paravalvular leakage,” *Med. Biol. Eng. Comput.*, vol. 57, no. 10, pp. 2129–2143, Oct. 2019, doi: 10.1007/s11517-019-02012-y.
[60] C. Dowling *et al.*, “Patient-Specific Computer Simulation of Transcatheter Aortic Valve Replacement in Bicuspid Aortic Valve Morphology,” *Circ. Cardiovasc. Imaging*, vol. 12, no. 10, 2019, doi: 10.1161/CIRCIMAGING.119.009178.

[61] S. Khodaei *et al.*, “Personalized intervention cardiology with transcatheter aortic valve replacement made possible with a non-invasive monitoring and diagnostic framework,” *Sci. Rep.*, vol. 11, no. 1, Dec. 2021, doi: 10.1038/s41598-021-85500-2.
[62] S. Khodaei, R. Sadeghi, P. Blanke, J. Leipsic, A. Emadi, and Z. Keshavarz-Motamed, “Towards a non-invasive computational diagnostic framework for personalized cardiology of transcatheter aortic valve replacement in interactions with complex valvular, ventricular and vascular disease,” *Int. J. Mech. Sci.*, vol. 202–203, Jul. 2021, doi: 10.1016/j.ijmecsci.2021.106506.
[63] C. Capelli *et al.*, “Patient-specific simulations of transcatheter aortic valve stent implantation,” *Med. Biol. Eng. Comput.*, vol. 50, no. 2, pp. 183–192, Feb. 2012, doi: 10.1007/s11517-012-0864-1.
[64] H. A. Dwyer, P. B. Matthews, A. Azadani, L. Ge, T. S. Guy, and E. E. Tseng, “Migration forces of transcatheter aortic valves in patients with noncalcific aortic insufficiency,” *Journal of Thoracic and Cardiovascular Surgery*, vol. 138, no. 5, pp. 1227–1233, 2009, doi: 10.1016/j.jtcvs.2009.02.057.
[65] T. Sirset-Becker *et al.*, “Modeling of valve-in-valve transcatheter aortic valve implantation after aortic root replacement using a 3-dimensional artificial intelligence algorithm,” *Journal of Thoracic and Cardiovascular Surgery*, vol. 169, no. 6, pp. 1684-1693.e3, Jun. 2025, doi: 10.1016/j.jtcvs.2024.06.024.
